# Hot Schrödinger cat states

**DOI:** 10.1126/sciadv.adr4492

**Published:** 2025-04-04

**Authors:** Ian Yang, Thomas Agrenius, Vasilisa Usova, Oriol Romero-Isart, Gerhard Kirchmair

**Affiliations:** ^1^ University of Innsbruck, Institute for Experimental Physics, 6020 Innsbruck, Austria.; ^2^ Institute for Quantum Optics and Quantum Information, Austrian Academy of Sciences, 6020 Innsbruck, Austria.; ^3^ University of Innsbruck, Institute for Theoretical Physics, 6020 Innsbruck, Austria.; ^4^ ICFO—Institut de Ciencies Fotoniques, The Barcelona Institute of Science and Technology, Castelldefels, 08860 Barcelona, Spain.; ^5^ ICREA, Passeig Lluis Companys 23, 08010 Barcelona, Spain.

## Abstract

The observation of quantum phenomena often necessitates sufficiently pure states, a requirement that can be challenging to achieve. In this study, our goal is to prepare a nonclassical state originating from a mixed state, using dynamics that preserve the initial low purity of the state. We generate a quantum superposition of displaced thermal states within a microwave cavity using only unitary interactions with a transmon qubit. We measure the Wigner functions of these “hot” Schrödinger cat states for an initial purity as low as 0.06. This corresponds to a cavity mode temperature of up to 1.8 kelvin, 60 times hotter than the cavity’s physical environment. Our realization of highly mixed quantum superposition states could be implemented with other continuous-variable systems, e.g., nanomechanical oscillators, for which ground-state cooling remains challenging.

## Teaser

A thermal initial state with low quantum purity is put into a superposition of displacements, while retaining its entropy.

## INTRODUCTION

The quantum superposition principle allows us to prepare a system in a superposition of two arbitrary states. The paradigmatic example is the superposition of two coherent states, which are pure states with Heisenberg-limited quantum fluctuations (*1*). While the superposition of coherent states is typically called a Schrödinger cat state, in Schrödinger’s original thought experiment, the cat—a body-temperature and out-of-equilibrium system—is prepared in a superposition of two mixed states dominated by classical fluctuations (*2*).

**Fig. 1.**
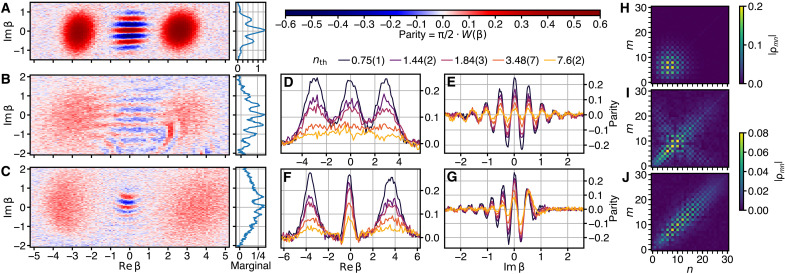
Wigner function measurement results. (**A**) Cold Schrödinger cat prepared using the qcMAP protocol with the heat bath disconnected (*n*_th_ = 0.03, P=0.94). (**B** and **C**) Hot Schrödinger cat states prepared from an initial thermal state with *n*_th_ = 3.48(7) using the ECD (B) and qcMAP (C) protocols. Also displayed in (A) to (C) are the marginal distributions obtained by summation along the Re {β} axis. To increase the visibility of small parity values, the color brightness changes nonlinearly across the color bar. (**D** to **G**) Linecuts of the Wigner function along the coordinate axes in the ECD (D and E) and qcMAP (F and G) protocols with *n*_th_ of the initial state as indicated in the legend. (**H** to **J**) Fock density matrices reconstructed from the Wigner measurement data in (A) to (C), respectively. (I) and (J) share the lower color bar. The matrix elements are given by $\rho_{\text{mn}}=\langle m|\hat{\rho }|n\rangle$.

Experimental demonstrations of Schrödinger cat states typically focus on a continuous-variable degree of freedom, such as the one-dimensional motion of a particle in a harmonic potential (*3*) or a single electromagnetic field mode in a cavity (*4*). The cat states are usually prepared by applying a given “cat state protocol” to an initial vacuum Fock state ${\hat{\rho }}_{0}=|0\rangle \langle 0|$, created by cooling the system to its ground state. This results in a pure quantum state that can be written as $|\alpha \rangle +e^{\mathrm{i\phi}}|-\alpha \rangle$, where ∣±α〉 are coherent states of the continuous-variable system with complex amplitude α and relative phase ϕ. We hereafter call this a “cold” Schrödinger cat state. It has an emblematic Wigner function exhibiting an interference pattern (see Fig. 1A).

One could ask what type of state would be prepared if the same cat state protocol is applied to an initial thermal state ${\hat{\rho }}_{T}$ with a finite average thermal excitation number *n*_th_ (*5*–*10*). Would these “hot” Schrödinger cat states exhibit quantum features given that (i) the purity of the initial state $P=\text{tr}\left[{\hat{\rho }}_{T}^{2}\right]=1/\left(2n_{\text{th}}+1\right)$ is substantially less than 1 for large *n*_th_, and (ii) we consider cat state protocols that do not remove entropy from the system? In other words, can a highly mixed state exhibit unambiguous quantum features?

In this article, we experimentally show that these hot Schrödinger cat states exhibit quantum features despite being highly mixed. More precisely, we implement two unitary protocols, previously used to prepare cold cats (*11*, *12*), on initial states with a nonzero *n*_th_. We vary *n*_th_ of the initial states up to 7.6 (2) [corresponding to P=0.062(2)] and perform direct Wigner function measurements on the final states. The created hot Schrödinger cat states show Wigner-negative interference patterns for all investigated values of *n*_th_ in the initial state (Fig. 1, B to G).

## RESULTS

Our experimental platform is a circuit quantum electrodynamics (cQED) setup (*13*). The hot cat states are prepared in a microwave cavity mode, which is well described as a quantum harmonic oscillator. The cavity mode is coupled to a two-level system that is used to prepare the cat states. The setup is placed inside a dilution refrigerator and cooled to a temperature of 30 mK (see fig. S1 for the full experimental schematic). The cavity is a high-coherence λ/4 post cavity, made up of high-purity niobium, with a resonance frequency $\omega_{c}/2\pi =4.545$ GHz (*14*), and a relaxation time $T_{1,c}=110\left(2\right)$ μs. For the two-level system, we use a transmon qubit with a resonance frequency $\omega_{q}/2\pi =5.735$ GHz, a qubit lifetime $T_{1}=31.0\left(4\right)$ μs, and a coherence time $T_{2}^{*}=12.5\left(4\right)$ μs. The cavity-qubit interaction is dispersive with the dominant Hamiltonian term $\hat{H}=-\hbar \chi_{\text{qc}}{\hat{c}}^{†}\hat{c}|e\rangle \langle e|$. Here, ${\hat{c}}^{†}$ and $\hat{c}$ are the cavity mode creation and annihilation operators, ∣e〉 is the excited state of the qubit, $\chi_{\text{qc}}/2\pi =1.499\left(3\right)$ MHz is the dispersive shift, and ℏ is the reduced Planck’s constant. Qubit state measurements are performed through an additional dispersively coupled microstrip resonator with frequency $\omega_{r}/2\pi =7.534$ GHz. This setup allows for direct measurements of the cavity Wigner function $W\left(\beta \right)\equiv 2\langle \hat{D}\left(\beta \right)\hat{\Pi }{\hat{D}}^{†}\left(\beta \right)\rangle /\pi$ (*15*). Here, β is a complex parameter, $\hat{D}\left(\beta \right)$ is the cavity displacement operator, and $\hat{\Pi }$ is the cavity parity operator (*16*). We calibrate the Wigner function measurement by preparing a single photon Fock state in the cavity (fig. S2).

The thermal initial state of the cavity mode, hotter than its environment, is created by equilibrating the cavity mode with a heat bath in the form of filtered and amplified Johnson-Nyquist noise from a 50-ohm resistor. The heat bath is then disconnected (to prevent it from causing additional decoherence) and the cat state preparation commences immediately. The state preparation and measurement protocols take up to 1.9 μs, which is much faster than the cavity relaxation time $T_{1,c}$. Thus, there is neither cooling nor heating of the cavity mode during the protocols. (In fig. S4, we additionally report results from running the experiment with the heat bath left connected during the entire protocol.) To verify that the produced initial state is thermal, we characterize the photon statistics of the cavity state with the added noise via qubit spectroscopy (*17*), which also allows us to relate the noise power to *n*_th_ (fig. S5).

**Fig. 2.**
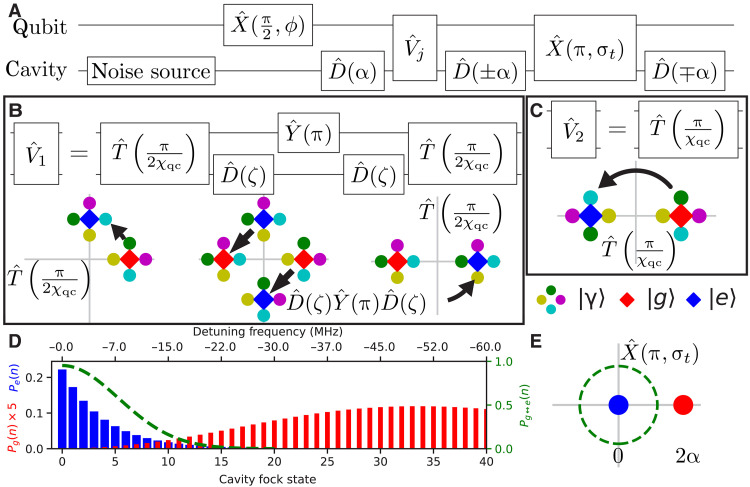
Hot cat state generation protocol. (**A**) Quantum circuit diagram of hot cat state generation sequence. For ECD (qcMAP), *j* = 1 (*j* = 2), and the displacements use the lower (upper) sign. $\hat{X}\left(\pi /2,\phi \right)$ is a qubit π/2 pulse with phase ϕ, and $\hat{X}\left(\pi ,\sigma_{t}\right)$ is the disentanglement pulse. (**B**) Definition of ${\hat{V}}_{1}$ and visualization of its action in the joint cavity-qubit phase space. $\hat{T}\left(t\right)$ denotes free evolution for time *t*, $\hat{Y}\left(\pi \right)$ is a qubit π pulse, and ζ=−(1+i)α/2. Cavity states are entangled with the qubit states whose marker they touch. The arrows illustrate how the total state evolves under the indicated operator. (**C**) Definition and visualization of ${\hat{V}}_{2}$. (**D**) Qubit-conditional cavity Fock state distributions $P_{q}\left(n\right)=\langle n|\langle q|\hat{\rho }|q\rangle |n\rangle$ (q∈{g,e}) of the total state before the $\hat{X}\left(\pi ,\sigma_{t}\right)$ operation. Here, α = 3 and *n*_th_ = 3.5. The dashed line shows $P_{g↔e}\left(n\right)$ (defined in the main text) with σ_*t*_ = 20 ns. (**E**) The choice of σ_*t*_ corresponds to the choice of a radius in the cavity-qubit phase space within which the qubit state is flipped with a certain probability. At this stage of the protocol, the ∣g〉 branch is displaced by 2α.

The two protocols used to prepare the hot cat states from the thermal initial state are adaptations of two protocols known in the cQED community as the echoed conditional displacement (ECD) (*11*) and qcMAP (*12*) protocols. The quantum circuit diagrams for the protocols we use are shown in Fig. 2A. To illustrate the state generation, we decompose the initial thermal state ${\hat{\rho }}_{T}$ in a basis of cavity coherent states $|\gamma \rangle =\hat{D}\left(\gamma \right)|0\rangle$ with γ being a complex parameter. This lets us discuss the action of the protocols on the state ∣γ〉∣g〉, where ∣g〉 is the qubit ground state, and then obtain the protocol’s action on the actual initial state ${\hat{\rho }}_{T}⊗|g\rangle \langle g|$ by averaging over γ in the explicit decomposition relation ${\hat{\rho }}_{T}={\left(\pi n_{\text{th}}\right)}^{-1}\int d^{2}\gamma \;\text{exp}\left\{-{|\gamma |}^{2}/n_{\text{th}}\right\}|\gamma \rangle \langle \gamma |$ (*1*). A pictorial version of this argument represents the thermal state as a “compass” of four slightly displaced coherent states, γ={ϵ,iϵ,−ϵ,−iϵ} for a small ϵ > 0, and tracks their motion in phase space during the protocol (Fig. 2, B and C). The first operation sequence prepares the qubit in the superposition state $|g\rangle +e^{\mathrm{i\phi}}|e\rangle$, where ϕ is a controllable phase shift, and the cavity in the displaced state ∣γ+α〉. Next, the cavity-qubit state becomes entangled through time evolution under the dispersive interaction $\hat{H}$, as illustrated in Fig. 2 (B and C) for ECD and qcMAP, respectively. The qcMAP protocol has an uninterrupted time evolution, while the ECD protocol has additional displacements and a qubit echo pulse inserted at half the evolution time. At the end of the time evolution, we have created the state $e^{\mathrm{i\phi}}|\mathrm{i\gamma}-\alpha \rangle |g\rangle +|\mathrm{i\gamma}+\alpha \rangle |e\rangle$ with the ECD protocol (Fig. 2B) and the state $|\gamma +\alpha \rangle |g\rangle +e^{\text{iϕ}}|-\gamma -\alpha \rangle |e\rangle$ with the qcMAP protocol (Fig. 2C). The final three operations in Fig. 2A disentangle the qubit from the cavity. We center the ∣e〉 branch with a displacement −α for ECD and α for qcMAP. Next, we apply a qubit π pulse selective to the ∣e〉 branch only. Last, we invert the previous displacement. The selective π pulse has a Gaussian envelope with standard deviation in time σ_*t*_. This induces a Fock number–dependent qubit flip probability $P_{g↔e}\left(n\right)=\text{exp}\left\{-{\left(\chi_{\text{qc}}\sigma_{t}n\right)}^{2}\right\}$ (*18*, *19*). In contrast to the known qcMAP and ECD protocol, where only the qubit state that is entangled with the cavity zero Fock state is affected, here we choose σ_*t*_ so that this probability is large for the nondisplaced thermal state but small for the displaced thermal state (Fig. 2D). This requires that α is chosen large enough so that the Fock number distributions of the initial thermal state and the thermal state displaced by 2α do not overlap (roughly, ${|\alpha |}^{2}>n_{\text{th}}+1$). In a phase-space picture, we flip the qubit only for phase-space points within a finite radius of the origin (Fig. 2E).

We run our experiment with an initial thermal excitation number of *n*_th_ = 0.75(1), 1.44(2), 1.84(3), 3.48(7), and 7.6(2), corresponding, respectively, to purities of P=0.400(3), 0.258(3), 0.214(3), 0.126(2), and 0.062(2). We use α = 3, set ϕ = 0, and use a disentanglement pulse width of σ_*t*_ = 20 ns. Figure 1 (B and C) shows Wigner function measurements on a grid of phase-space points β for *n*_th_ = 3.48. Figure 1 (D to G) shows Wigner function measurements along the Re{β} and Im{β} axes as *n*_th_ is varied. Figure 1 (H to J) shows Fock density matrices reconstructed from the Wigner measurements in Fig. 1 (A to C); see Materials and Methods for the reconstruction method. In fig. S3, we additionally show Wigner function measurements for *n*_th_ = 1.84(3). While the ECD and qcMAP protocols are known to prepare equivalent cold cats, we observe that they lead to distinct outcomes when applied to thermal initial states; compare (B) and (C) in Fig. 1. In both cases, the Wigner functions have two separated Gaussians centered at α and −α, associated with the classical probability distribution of the displaced thermal initial states. Centered between these is an interference pattern of oscillations with negative values, which produces an interference pattern in the marginal distribution along Im{β}. In the ECD state, the envelope of the interference pattern grows in radius and decreases in amplitude with *n*_th_ similar to the displaced thermal states themselves (Fig. 1, D and E). In the qcMAP state, the envelope shrinks with increasing *n*_th_, but its amplitude decreases more slowly than the displaced thermal states (Fig. 1, F and G). The data for all prepared states show clear negative values in the interference pattern regardless of their *n*_th_.

**Fig. 3.**
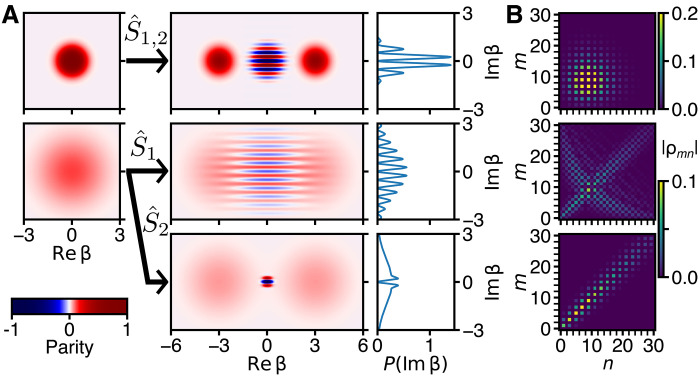
Hot Schrödinger cat states in theory. (**A**) Left: Plots of the thermal state Wigner function $W_{T}\left(\beta \right)$ with $n_{\text{th}}=0$ (top) and $n_{\text{th}}=3.5$ (middle). To increase the visibility of the hotter state, the color brightness changes nonlinearly across the color bar (bottom). Center: Cat state Wigner functions $W_{1,2}\left(\beta \right)$, which result from applying the operators ${\hat{S}}_{1,2}$ with = 3.5 and ϕ=π to the initial states on the left according to the arrows. Right: Marginal probability distributions obtained from the cat state Wigner functions by integrating along the Re{β} axis. (**B**) Absolute values of the Fock density matrix elements corresponding to the hot cat states displayed on the same row. The middle and bottom panels share the lower color bar. The matrix elements are given by $\rho_{\text{mn}}=\langle m|\hat{\rho }|n\rangle$.

These results can be understood as follows. Under ideal conditions, the ECD and qcMAP protocols are equivalent to the application of two different quantum operators, namely, ${\hat{S}}_{1}\equiv \left[\hat{D}\left(\alpha \right)+e^{i\phi }\hat{D}\left(-\alpha \right)\right./\sqrt{2}$ (ECD) and ${\hat{S}}_{2}\equiv \left[1+e^{\mathrm{i\phi}}\hat{\Pi }\right]\hat{D}\left(\alpha \right)/\sqrt{2}$ (qcMAP), to the cavity initial state ${\hat{\rho }}_{T}$. (The ECD protocol is more generally equivalent to the operator ${\hat{S}}_{1}^{\prime }\equiv {{\hat{S}}_{1}|}_{\phi \to \phi +2{|\alpha |}^{2}}i^{{\hat{c}}^{†}\hat{c}}$, but these differences vanish when the initial state is thermal and the geometric phase $2{|\alpha |}^{2}$ is cancelled by ϕ; see Materials and Methods.) The Wigner functions prepared from ${\hat{\rho }}_{T}$ by the operators ${\hat{S}}_{1,2}$ are $W_{1,2}\left(\beta \right)\equiv \frac{1}{2}\left[W_{T}(\beta -\alpha )+W_{T}\left(\beta +\alpha \right)+C_{1,2}\left(\beta \right)\right]$, where $W_{T}\left(\beta \pm \alpha \right)$ are the Wigner functions of the left- and right-displaced thermal states, and the third term, which represents the quantum superposition property of the state, is different for the two preparations: $C_{1,2}\left(\beta \right)=2\text{cos}\left(4\text{Im}\;\left\{\alpha^{*}\beta \right\}+\phi \right)f_{1,2}\left(\beta \right)$. For ECD, $f_{1}\left(\beta \right)\equiv W_{T}\left(\beta \right)=2Pe^{-2P{|\beta |}^{2}}/\pi$ is the thermal initial state. For qcMAP, $f_{2}\left(\beta \right)\equiv 2e^{-2{|\beta |}^{2}/P}/\pi$ is related to the characteristic function (*20*) of the initial state. We plot examples of $W_{1,2}\left(\beta \right)$ in Fig. 3A. As illustrated there, when $n_{\text{th}}\to 0$ (P→1), $f_{1,2}\left(\beta \right)$ become identical and $W_{1,2}\left(\beta \right)$ both become equal to the cold cat Wigner function. When $n_{\text{th}}>0$, the phase-space radius of $f_{1}\left(\beta \right)$ grows at the same rate as the phase-space radii of $W_{T}\left(\beta \pm \alpha \right)$, while the phase-space radius of $f_{2}\left(\beta \right)$ shrinks. For both states, the Im {β} marginal probability distribution contains interference fringes with period π/2α and full contrast independently of $n_{\text{th}}$. We remark that for ϕ=nπ with *n* integer, $W_{2}\left(0\right)$ is always saturated to the Wigner function upper/lower bounds ±2/π, corresponding to parity values $\langle \hat{\Pi }\rangle =\pm 1$, independently of $n_{\text{th}}$. Realizing this saturation of parity could be useful for hardware-efficient encoding in bosonic qubit states in the presence of finite mode temperature. For example, in (*21*, *22*), the stabilization process via two-photon dissipation or parametric pumping is parity preserving. An initial thermal cavity state will reduce the prepared state fidelity. This can be overcome by first using the qcMAP operator before stabilization. The sharp saturation of parity in a small phase-space volume in the qcMAP state could also be explored for applications in quantum metrology. For comparison with Fig. 1 (H to J), we display the Fock density matrices of the ideal hot cat states in Fig. 3B.

**Fig. 4.**
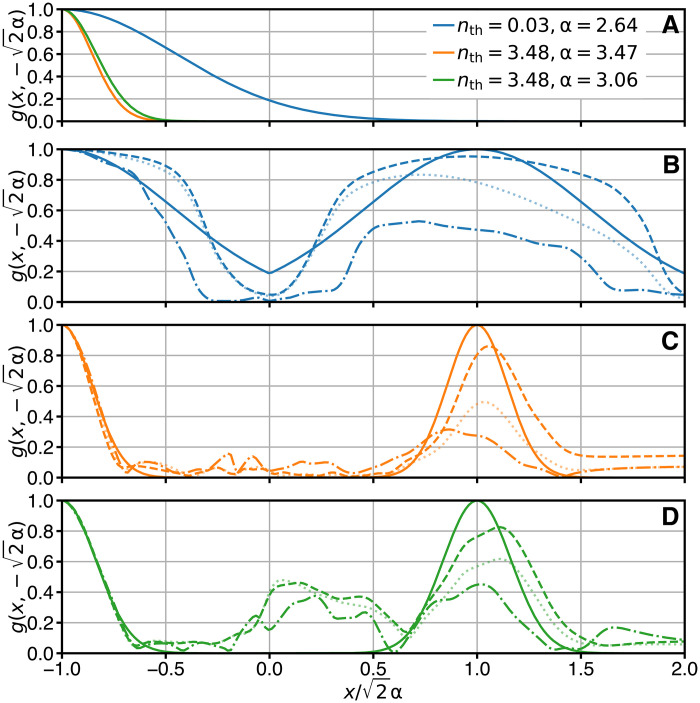
Ideal, reconstructed, and numerically simulated coherence functions. (**A**) Sections of the theoretical thermal state coherence function (given in the text) with values of α and *n*_th_ as indicated in the panel legend. (**B** to **D**) Sections of the coherence function for the (B) cold cat, (C) hot qcMAP cat, and (D) hot ECD cat states along the line from $\left(x_{1},x_{2}\right)=\sqrt{2}\alpha \left(-1,-1\right)$ to $\left(x_{1},x_{2}\right)=\sqrt{2}\alpha \left(2,-1\right)$. The solid lines indicate the ideal states with the values of *n*_th_ and α given in the legend in (A). The dash-dotted line is computed from the density matrix reconstruction of the measured experimental data (Fig. 1, H to J). Dashed lines are computed from density matrix reconstructions performed on the output of the ab initio numerical model of the state preparation discussed in the text. The dotted semitransparent lines are included to demonstrate the output of the numerical model if additional cavity dephasing is added (see Discussion).

To highlight the quantum superposition nature of the hot cat states, we consider the coherence function $g\left(x_{1},x_{2}\right)\equiv |\langle x_{1}|\hat{\rho }|x_{2}\rangle |/\sqrt{\langle x_{1}|\hat{\rho }|x_{1}\rangle \langle x_{2}|\hat{\rho }|x_{2}\rangle }$ of the prepared states. Here, $x_{1,2}$ are eigenvalues and $|x_{1,2}\rangle$ are eigenkets of the dimensionless quadrature operator $\hat{x}\equiv \left(\hat{c}+{\hat{c}}^{†}\right)/\sqrt{2}$, which is defined in analogy to the position operator of a mechanical harmonic oscillator. The coherence function compares the off-diagonal elements of the density matrix to their maximum possible value and, thus, is upper-bounded as $g\left(x_{1},x_{2}\right)\leq 1$. The value of $g\left(\sqrt{2}\alpha ,-\sqrt{2}\alpha \right)$ quantifies, on a continuous scale from 0 to 1, whether the oscillator is observed to be in a quantum superposition of being displaced by α and −α (the $\sqrt{2}$ factor is due to the definition of the position quadrature operator $\hat{x}$). This value is also directly related to the contrast of the interference fringes in the marginal distributions (*23*). We reconstruct the coherence functions of our experimentally prepared states along the line from $\left(x_{1},x_{2}\right)=\sqrt{2}\alpha \left(1,1\right)$ to $\sqrt{2}\alpha \left(1,-1\right)$ via the reconstructed density matrices of Fig. 1 (H to J). We present the results in Fig. 4 along with theoretical curves. Consider first the ideal case, represented by solid lines in Fig. 4. For both states prepared by ${\hat{S}}_{1,2}$, $g\left(x_{1},x_{2}\right)=e^{-{\left(|x_{1}|-|x_{2}|\right)}^{2}/2\xi_{\text{th}}^{2}}$ along the considered line. [Away from this line, the coherence functions of $W_{1,2}\left(\beta \right)$ are different; see fig. S8.] The quantity $\xi_{\text{th}}=\sqrt{2P/\left(1-P^{2}\right)}\approx 1/\sqrt{n_{\text{th}}}$ is the thermal coherence length. When $x_{1}\approx x_{2}$, the cat state coherence functions behave like those of thermal states, $e^{-{|x_{1}-x_{2}|}^{2}/2\xi_{\text{th}}^{2}}$, with the corresponding values of *n*_th_. As *n*_th_ increases, ξ_th_ decreases and the thermal state coherence function decays faster with $|x_{1}-x_{2}|$ (Fig. 4A). The peak at $x_{1}=-x_{2}=\sqrt{2}\alpha$ in the cat state coherence functions (solid lines in Fig. 4, B to D), which is not present in the thermal state coherence function, indicates the quantum superposition nature of these states. In the ideal case, this peak saturates to the upper bound $g\left(\sqrt{2}\alpha ,-\sqrt{2}\alpha \right)=1$, with the value of *n*_th_ only affecting how narrow this extra peak is. The data (dash-dotted lines in Fig. 4, B to D) show similar behavior to the ideal case, but deviate both due to the limitations of the density matrix reconstruction method and due to the differences between the experiment and the ideal case. We discuss the latter further below. Here, we note that the cat state data always display a peak around $\left(x_{1},x_{2}\right)=\sqrt{2}\alpha \left(1,-1\right)$, the height of which is not substantially affected by the value of *n*_th_.

## DISCUSSION

We emphasize that the additional peak in the cat state coherence function comes from the unitary operations applied to the initial state (i.e., the cat creation operators ${\hat{S}}_{1,2}$). These operations thus generate coherence not present in the initial state. Both protocols generate the same amount of coherence (as shown by the coherence function), resulting in equally coherent superpositions of mixed states, despite the parity of the hot ECD cat state being lower than the hot qcMAP state [as shown by the Wigner functions at the origin $W_{1,2}\left(0\right)$]. The additional peak in the coherence function is also generated in experiments that observe interference patterns from superpositions of thermal clouds of atoms prepared using Bragg diffraction (*24*) or Stern-Gerlach interferometry (*25*). In contrast, the additional peak is not present in the state obtained by sending a thermal state through a double slit grating [see, e.g., (*26*)] or in a completely dephased cold cat state, namely, ${\hat{\rho }}_{\text{dephased}}=\frac{1}{2}\left(|\alpha \rangle \langle \alpha |+|-\alpha \rangle \langle -\alpha |\right)$ [see, e.g., (*27*)]. Note that ${\hat{\rho }}_{\text{dephased}}$ has purity 1/2 but a completely positive Wigner function [$W_{1,2}\left(\beta \right)$ with $C_{1,2}\left(\beta \right)=0$], whereas we experimentally prepare states with negative Wigner functions down to purities of 0.06.

The measured Wigner functions (Fig. 1) deviate from the ideal Wigner functions $W_{1,2}\left(\beta \right)$ (Fig. 3A) mainly due to qubit operation imperfections, perturbative Hamiltonian nonlinearities, and cavity-qubit system decoherences. Through the reconstructed density matrices, we estimate the fidelity of the experimentally prepared states to the ideal cat states $W_{1,2}\left(\beta \right)$ to be 0.8 for the cold cat and 0.7 for the hot cats. The loss in fidelity can be partly accounted for by qubit coherence time (3%), cavity lifetime (5%), and qubit population (2%). To determine the role of other factors, we use our characterization of the experiment (fig. S6 and table S1) to construct an ab initio model of the state preparation protocols that includes the nonlinearities, the shape and timings of the qubit pulses, and decoherence during state preparation. The Wigner function measurement is modeled as a projective measurement that includes the effects of residual cavity-qubit entanglement due to pulse imperfections (see Materials and Methods). We find that the model reproduces most of the imperfections seen in the data (see figs. S9 and S10). The main remaining discrepancy is that the model states are more coherent than the reconstructed states, as can be seen by comparing dashed and dash-dotted lines in Fig. 4. In this comparison, the model states are expected to show only half the decoherence when compared to the reconstructed states, because the model excludes decoherence during the measurement sequence. The remaining extra decoherence is due to uncharacterized additional loss channels, most likely cavity dephasing, whose presence is indicated by cat parity lifetime measurements (fig. S7). We leave the exact characterization of this noise for future work. Adding a small amount of dephasing to the ab initio model improves the fidelity to the reconstructed states as well as the qualitative agreement [for illustration, see the dotted curve in Fig. 4, which has an added cavity dephasing rate of 1/(80 μs)].

By running the ab initio model and toggling each feature of the model, such as operation imperfections or Hamiltonian nonlinearities, between being included and excluded, we attribute each discrepancy between the measured and ideal Wigner functions to a cause (figs. S11 to S13). Here, we summarize the conclusions and further details are given in section S4. The leading perturbative Hamiltonian terms H^′=−12(Kc+χ′qc∣e〉〈e∣)cˆ†cˆ†cˆcˆ [$K_{c}/2\pi =4.9\left(1\right)$ kHz, $\chi \prime_{\text{qc}}/2\pi =12.8\left(9\right)$ kHz] cause a smearing and bending distortion of the Wigner functions, which is similar to that observed in previous experiments (*28*). The 20-ns width of the disentanglement pulse causes left-right asymmetries of the displaced thermal states as well as bending distortions of the fringes. In particular, the nonlinearities and finite pulse widths together cause the qcMAP linecuts (Fig. 2D) to deviate from those predicted by $W_{2}\left(\beta \right)$, with an *n*_th_-dependent reduction of the maximum of $C_{2}\left(\beta \right)$. The extra fringes in the ECD state Wigner function as compared to $W_{1}\left(\beta \right)$ are caused by instrumentation limitations to the minimum qubit pulse width that we can achieve (Gaussian standard deviation of 6 ns).

We have demonstrated and characterized the preparation of quantum superposition states directly from thermally excited initial states using only unitary dynamics. Preparing “hotter” (larger initial thermal occupation number) cat states in our setup requires using larger cavity displacements so that the ancillary qubit can be disentangled from the cavity in the final step of the protocol. Limitations eventually appear due to the finite coherence time, finite pulse width, and perturbative nonlinearities in our experiment. In this context, we note that state-of-the-art cQED setups capable of cold cat states with α = 32 were recently reported (*29*). In other systems, limitations arise in the measurement of the prepared state rather than in its preparation. For example, the narrow thermal coherence length of the hot cats leads to increased time-of-flight requirements to see interference fringes in a Bragg (*24*) or half–Stern-Gerlach interferometer (*25*) and leads to increased recombination precision requirements in a full-loop Stern-Gerlach interferometer (*30*), compared to cold cats. Nevertheless, under ideal conditions including ideal measurement precision, standard quantum-mechanical theory predicts no upper limit on and no loss of contrast due to the thermal occupation number of a hot cat state (*5*–*10*). Hot Schrödinger cat states are, in principle, realizable in any continuous-variable quantum system. This is particularly relevant for systems where long coherence times have been achieved but ground-state cooling is not (yet) available. Specific examples include nanomechanical systems such as carbon nanotubes (*31*), and levitated magnetic (*32*, *33*) and electrostatically trapped dielectric particles (*34*–*37*). Our work highlights both opportunities and challenges associated with designing protocols for the observation of quantum phenomena that do not require ground-state cooling (*38*, *39*).

## MATERIALS AND METHODS

### Fabrication

The transmon qubit and readout resonator were patterned by electron-beam lithography (Raith eLINE Pllus 30 kV) on a bilayer resist [1 μm MMA (8.5) EL13 and 0.3 μm of 950 PMMA A4]. The substrate started from a 2-inch (5.08-cm) sapphire wafer that was first piranha cleaned before processing. To prevent charging of the substrate, a thin gold layer was sputtered on top of the PMMA. After lithography, this gold layer was etched in a solution of Lugol (5% potassium iodide) and deionized (DI) water in a ratio of 1:15, before being washed in DI water and developed in a 3:1 solution of isopropyl alcohol and water. In the next step, two layers of aluminum (25 and 50 nm) were evaporated onto the sample using a Plassys MEB550S electron-beam evaporator. A controlled oxidation step (5 mbar for 5.5 min) was carried out in between the deposition of the two aluminum layers. Subsequently, the qubit chip was laser diced, and the resist layer was lifted off. The sample chips were thermalized by a copper clamp. More details about the experimental setup can be found in section S1.

### Density matrix and coherence function reconstruction and fidelity estimates

To reconstruct the density matrices $\hat{\rho }$ of the prepared states, we compute the matrix elements in the Fock basis $\rho_{\mathrm{mn}}$ as $\rho_{\mathrm{mn}}=\langle m|\hat{\rho }|n\rangle =2\int d^{2}\beta \;W\left(\beta \right)\langle m|\hat{\Pi }\left(\beta \right)|n\rangle$_,_ where $\hat{\Pi }\left(\beta \right)=\hat{D}\left(\beta \right)\hat{\Pi }{\hat{D}}^{†}\left(\beta \right)$ and ∣n〉 is the *n*:th cavity Fock state. We approximate the integral as a Riemann sum on the phase-space grid of measured data for W(β). The matrix $\rho_{\mathrm{mn}}$ that we obtain from the data is not a density matrix, but rather a noisy estimate of the operator $\hat{\rho }=p_{g}{\hat{\rho }}_{g}-p_{e}{\hat{\rho }}_{e}$, where $p_{e}$ is the residual excited state qubit population after the state preparation protocol, $p_{g}=1-p_{e}$, and ${\hat{\rho }}_{g}$ (${\hat{\rho }}_{e}$) is the cavity state conditional on the qubit being in the ground (excited) state. The state we are seeking to reconstruct is ${\hat{\rho }}_{g}$. To extract the corresponding Fock matrix $\rho_{g}$ from the matrix $\rho =\left(\rho_{\mathrm{mn}}\right)$, we diagonalize ρ and then construct $\rho_{g}$ as the positive semidefinite part of ρ. From the reconstructed density matrix $\rho_{g}$, we compute the fidelity F of the considered state to a reference state $\hat{\rho }\prime$ as $F=(\text{Tr}\;\left\{\sqrt{\sqrt{\hat{\rho }\prime }{\hat{\rho }}_{g}\sqrt{\hat{\rho }\prime }}\right\})$2. We compute the coherence function by expressing ${\hat{\rho }}_{g}$ in the eigenbasis of $\hat{x}$. Formulas and further details of the steps described here are given in section S1.6.

### Derivation of equivalent operators for the ideal pulse sequences and the ideal Wigner functions

Here, we summarize a longer derivation that we give in sections S2.1 and S2.2. The ECD and qcMAP protocols are described in Hilbert space by two unitary operators ${\hat{U}}_{1,2}$ which are given in the circuit diagrams of Fig. 2. When applied to an initial state ${\hat{\rho }}_{0}⊗|g\rangle \langle g|$, where ${\hat{\rho }}_{0}$ is a general initial cavity state, the protocols result in total cavity-qubit states ${\hat{\rho }}_{f}={\hat{S}}_{j,\mathrm{gg}}{\hat{\rho }}_{0}{\hat{S}}_{j,\mathrm{gg}}^{†}⊗|g\rangle \langle g|+{\hat{S}}_{j,\mathrm{eg}}{\hat{\rho }}_{0}{\hat{S}}_{j,\mathrm{eg}}^{†}⊗|e\rangle \langle e|+\hat{\psi }$, where ${\hat{S}}_{j,\mathrm{gg}}=\langle g|{\hat{U}}_{j}|g\rangle$ and ${\hat{S}}_{j,\mathrm{eg}}=\langle e|{\hat{U}}_{j}|g\rangle$ for j∈{1,2}, and $\hat{\psi }$ represents coherence terms between ∣g〉 and ∣e〉. We identify $p_{g}=\text{Tr}\;\left\{{\hat{S}}_{j,\mathrm{gg}}{\hat{\rho }}_{0}{\hat{S}}_{j,\mathrm{gg}}^{†}\right\}$, ${\hat{\rho }}_{g}={\hat{S}}_{j,\mathrm{gg}}{\hat{\rho }}_{0}{\hat{S}}_{j,\mathrm{gg}}^{†}/p_{g}$, and similar expressions for $p_{e}$ and ${\hat{\rho }}_{e}$, so that ${\hat{\rho }}_{f}=p_{g}{\hat{\rho }}_{g}⊗|g\rangle \langle g|+p_{e}{\hat{\rho }}_{e}⊗|e\rangle \langle e|+\hat{\psi }$. Unitarity of ${\hat{U}}_{1,2}$ implies $p_{g}+p_{e}=1$. If $p_{g}=1$, then acting with ${\hat{U}}_{j}$ on the cavity-qubit system effectively applies the operator ${\hat{S}}_{j,\mathrm{gg}}$ to the cavity initial state while leaving the qubit in ∣g〉. From ${\hat{U}}_{1,2}$, one derives ${\hat{S}}_{1,\mathrm{gg}}=\frac{1}{\sqrt{2}}\hat{D}\left(\alpha \right)\left[{\text{ie}}^{i\left(\phi +{|\alpha |}^{2}\right)}\langle g|\hat{X}\left(\pi ,\sigma_{t}\right)|g\rangle \hat{D}\left(-2\alpha \right)-e^{-i{|\alpha |}^{2}}\langle g|\hat{X}(\pi ,\sigma_{t}\right)|e\rangle ]i^{{\hat{c}}^{†}\hat{c}}$, ${\hat{S}}_{2,\mathrm{gg}}=\frac{1}{\sqrt{2}}\hat{D}\left(-\alpha \right)\left[\langle g|\hat{X}\left(\pi ,\sigma_{t}\right)|g\rangle \hat{D}\left(2\alpha \right)+{\text{ie}}^{i\phi }\hat{\Pi }\langle g|\hat{X}(\pi ,\sigma_{t})|e\rangle \right]$, where $\langle g|\hat{X}\left(\pi ,\sigma_{t}\right)|e\rangle$ and $\langle g|\hat{X}\left(\pi ,\sigma_{t}\right)|g\rangle$ are operators on the cavity Hilbert space. If $\langle g|\hat{X}\left(\pi ,\sigma_{t}\right)|e\rangle {\hat{\rho }}_{0}={\hat{\rho }}_{0}$ while $\langle g|\hat{X}(\pi ,\sigma_{t})|g\rangle \hat{D}\left(\pm 2\alpha \right){\hat{\rho }}_{0}=\hat{D}\left(\pm 2\alpha \right){\hat{\rho }}_{0}$, then it follows that $p_{g}=1$. A necessary condition is that ${\hat{\rho }}_{0}$ has no overlap with itself when displaced by 2α. We then recover $\hat{S}{'}_{1}$ and ${\hat{S}}_{2}$ from ${\hat{S}}_{1,\mathrm{gg}}$ and ${\hat{S}}_{2,\mathrm{gg}}$ after dropping global phases and redefining ϕ to cancel relative phases. The stated conditions are the formal versions of the condition that the disentanglement pulse should flip the qubit state only for the cavity state entangled to ∣e〉. The ideal Wigner functions $W_{1,2}\left(\beta \right)$ follow directly from the Wigner representations of ${\hat{\rho }}_{g}$ when $p_{g}=1$.

### Numerical model

We simulate the ECD and qcMAP protocols using QuTiP version 4.7 (*40*). We model the time evolution operations $\hat{T}$ and the qubit and cavity-conditional qubit operations $\hat{X},\hat{Y}$ by numerically integrating the master equation describing the total cavity-qubit dynamics with parameters (system Hamiltonian, decay rates, and qubit driving Hamiltonian) as characterized in the experiment. Displacement operators and thermal initial states are implemented using QuTiP’s built-in functions. We model the Wigner function measurement as the expectation value of the observable $\hat{M}\left(\beta \right)\equiv \frac{2}{\pi }\hat{\Pi }\left(\beta \right)\left(|g\rangle \langle g|-|e\rangle \langle e|\right)$ in the final states of the protocols. This model accounts for the effect of the residual qubit ∣e〉 population after state preparation but neglects the influence of decoherence and nonlinearities during the measurement sequence (*15*). More details are given in section S3. Details on the subsequent investigation to study the effects of operation imperfections and Hamiltonian nonlinearities individually are given in section S4.

## Supplementary Materials

### This PDF file includes:

Sections S1 to S4 Figs. S1 to S13 Table S1 References
