# Supplementary material for: Hot Schrödinger cat states
Source: Sci Adv. 2025 Apr 4;11(14):eadr4492. doi: 10.1126/sciadv.adr4492 (PMC13109942; doi:10.1126/sciadv.adr4492)
Supplement: Supplementary file 1 — Sections S1 to S4 Figs. S1 to S13 Table S1 References [file sciadv.adr4492_sm.pdf]

Science Advances
AAAS

Supplementary Materials for

Hot Schrödinger cat states

Ian Yang *et al.*

Corresponding author: Gerhard Kirchmair, gerhard.kirchmair@uibk.ac.at

Sci. Adv. **11**, eadr4492 (2025)
DOI: 10.1126/sciadv.adr4492

This PDF file includes:

Sections S1 to S4
Figs. S1 to S13
Table S1
References

S1 Experiment

S1.1 Experimental Setup

The schematic of the experimental setup is shown in Figure S1. The high coherence cavity has a post length of 14.8 mm, inner radius of 2 mm and outer radius of 6.4 mm. This geometry gives a bare cavity frequency of approximately 4.5 GHz. The tunnel for the qubit chip has a diameter of 4 mm, which is a compromise between cavity mode leakage into the tunnel and qubit capacitance to ground. The cavity was made from high-purity niobium at the Institute for Quantum Optics and Quantum Information Innsbruck mechanical workshop. The manufacturing process used electro-discharge machining with a tungsten alloy electrode. The cavity was then etched with our collaborators at the Institute of Science and Technology, Vienna with the group of Prof. Johannes Fink. This process used a buffer chemical polishing etching solution of 1:1:1 hydrofluoric, nitric and phosphoric acid for one hour at 5 °C. Phosphoric acid was then slowly added to reach a ratio of 1:1:2 for another hour of polishing. Afterwards, the niobium cavity was rinsed heavily with deionized (DI) water. In total, this process removes approximately 150 µm of material.

The transmon qubit and readout resonator were patterned by electron-beam lithography (Raith eLINE Pllus 30 kV) on a bi-layer resist (1 µm MMA (8.5) EL13 and 0.3 µm of 950 PMMA A4). The substrate started from a 2-inch sapphire wafer that was first piranha-cleaned before processing. To prevent charging of the substrate, a thin gold layer was sputtered on top of the PMMA. After lithography, this gold layer was etched in a solution of Lugol (5 % potassium iodide) and DI water in a ratio of 1:15, before being washed in DI water and developed in a 3:1 solution of isopropyl alcohol and water. In the next step, two layers of aluminum (25 nm and 50 nm) were evaporated onto the sample using a Plassys MEB550S electron-beam evaporator. A controlled oxidation step (5 mbar for 5.5 min) was carried out in between the deposition of the two aluminum layers. Subsequently, the qubit chip was laser-diced, and the resist layer was lifted off. The sample chips were thermalized by a copper clamp. An additional aluminum sheet was used to cover the copper clamp to reduce losses due to the presence of the copper material.

The measurements were conducted in a Triton DU7-200 Cryofree dilution refrigerator system. The input coaxial cables were attenuated by 20 dB at the 4 K plate and 10 dB at the still plate. Finally, at the mixing chamber plate, the input signal was filtered by a K&L DC-12 GHz low pass filter and then attenuated by a 20 dB directional coupler followed by a thermalized cryogenic 20 dB attenuator and filtered by microtronics $4-8$ GHz bandpass filter. The experiment was done in reflection with a Quinstar double junction $4-8$ GHz circulator. Before and after the sample, the input and output signals passed through a home-built eccosorb filter. The input signal for the high coherence cavity was attenuated and filtered similarly, except at the base plate where a 10 dB thermalized cryogenic attenuator was used instead.

The output signal was filtered via a microtronics $4-8$ GHz bandpass filter, before passing through a quantum-limited parametric amplifier. Finally, the output signal was filtered by a K&L filter which was connected to two Quinstar isolators giving 40 dB isolation. The signal was

amplified at the 4 K plate by high electron mobility transistor (HEMT) amplifiers and again with room temperature amplifiers outside of the refrigerator.

Control of the thermal noise was done by amplifying and filtering the noise from a 50 ohm resistor. The added noise has a frequency spectrum shown in Figure S4A. The added noise power level was controlled by a digital attenuator. The setup allowed for a maximum of 60 dB of added thermal noise. A fast, home-built microwave switch was used to disconnect the cavity mode from this added noise. The switch has an open attenuation of 40 dB and has a rise and fall time of around 10 ns. To initialize the cavity state, the cavity mode was allowed to come into thermal equilibrium with the controlled noise environment for 1 ms. Afterwards, the microwave switch was opened and the state preparation and measurement started.

Leaving the microwave switch opened resulted in a thermal state in equilibrium with the residual thermal excitations of the setup ($n_{\rm th} = 0.0338(7)$), which was the coldest initial state we could achieve with this setup.

The samples were placed in a μ-metal shield which sat in a superconducting shield to protect the experiment against stray magnetic fields. The shield was filled with eccosorb foam for the absorption of any stray infrared photons.

The pulses for the high coherence cavity and readout resonator were generated by an arbitrary waveform generator (AWG), specifically the Operator X from Quantum Machines. These pulses were up-mixed with a local oscillator (LO) using a Marki microwave IQ mixer. The qubit pulses, on the other hand, were up-mixed through a double-super-heterodyne (*41*) setup employing two LOs and two single side-band mixers. These pulse generation setups also incorporated various amplifiers, filters, alternators, and fast microwave switches to achieve effective suppression of unwanted mixing products and to minimize leakage of LO signals. The signal from the refrigerator was down-mixed using the same readout LO and further amplified before being digitized by Operator X from Quantum Machines.

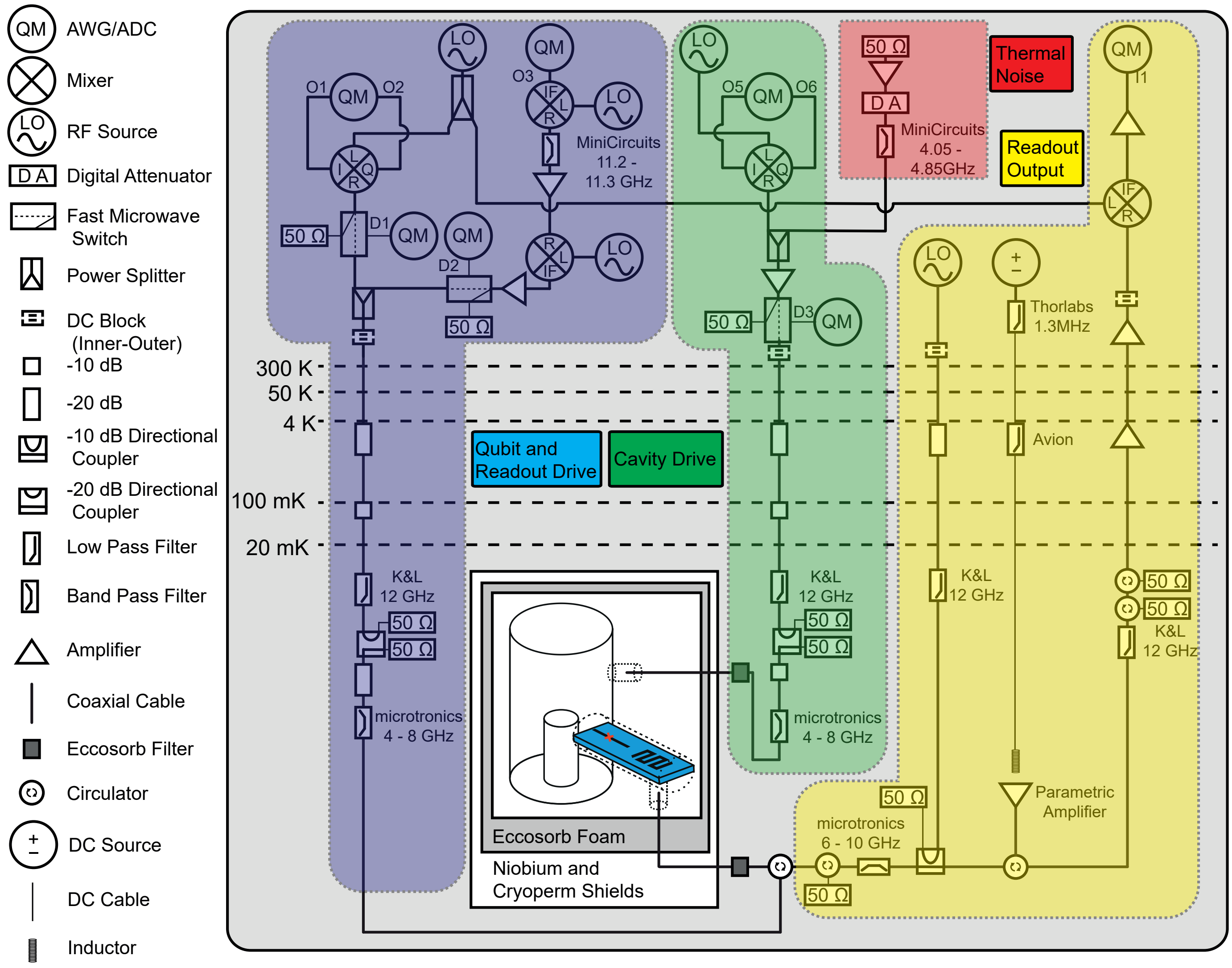

Fig. S1. Schematic of Experimental Wiring. The cavity and readout resonator were driven by an IQ mixing setup while the qubit tones were up-converted via a double-super-heterodyne setup. The qubit-resonator line had a total of 70 dB input line attenuation while the cavity had 60 dB. Cavity noise was added via amplifying Johnson-Nyquist noise at room temperature by a total of 60 dB and filtered with a MiniCircuits filter. The noise level was reduced by a digital attenuator. The readout tone was first amplified with a parametric amplifier before reaching the HEMTs. Noise from the output and amplifier pump lines were attenuated with isolators. Additional microwave and eccosorb filters were used to remove unwanted radiation from reaching the experiment. The setup was placed in superconducting and μ-metal shields and was surrounded by eccosorb foam.

S1.2 Calibration and Scaling of Wigner Function

The Wigner function measurement was calibrated by the measurement of a single photon Fock state. The single photon Fock state was prepared by using a blue sideband transition. This technique is similar to that used in ion traps (*42*) or atomic arrays (*43*). The measurement data, which has arbitrary units, was collected in the pulse quadrature variables I, Q, which also have arbitrary units (Figure S2A). To calibrate the measurement, we seek a linear map from the data space into phase space. We find this map by fitting the function $\chi_W^{-1} W_{|1\rangle}(\chi_I I + \mathrm{i}\chi_Q Q)$ to the Fock state measurement data, where χ_W, χ_I, and χ_Q are scaling constants, and $W_{|1\rangle}(\beta)$ is the Wigner function of the first Fock state (Figure S2B,C). Using the fitted scaling constants, we then map all other Wigner function measurement data into phase-space by constructing $\beta = \chi_I I + \mathrm{i}\chi_Q Q$ and $W(\beta) = \chi_W D(I, Q)$, where $D(I, Q)$ is the measurement data.

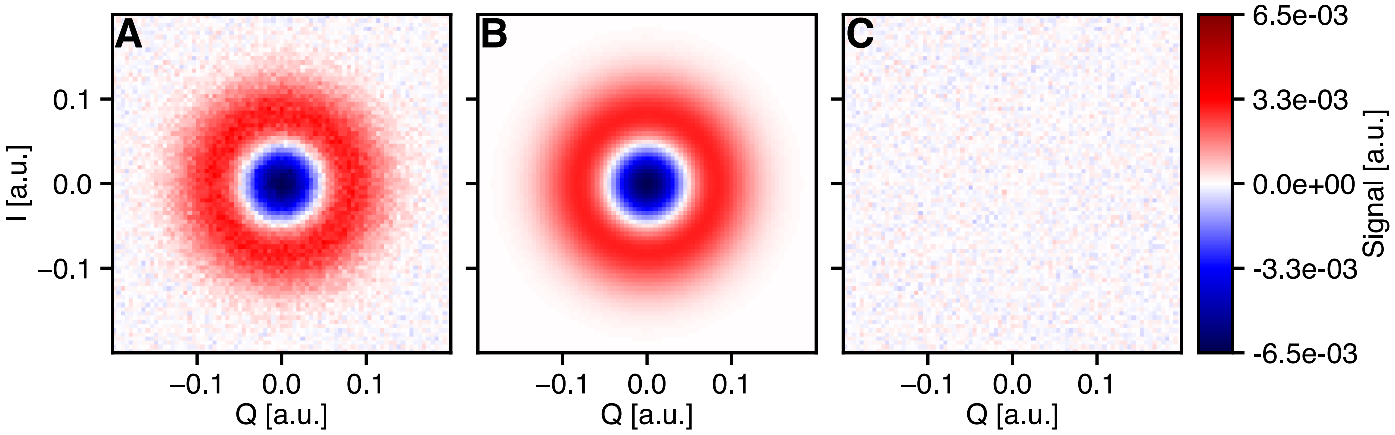

Fig. S2. Wigner Measurement and Fit of a Single Photon Fock State. (**A**) Measured data and (**B**) analytical fit of a $|1\rangle$ cavity Fock state in data space. (**C**) Residuals of fit. We use the parameters of the fit to construct a linear map from data space into phase space (Section S1.2).

S1.3 Additional Hot Cat Wigner Function Measurements

In Figure S3, we show additional Wigner function measurement maps done with the same protocol with an initial mean thermal cavity photon number of $n_{\mathrm{th}} = 1.84(3)$.

In Figure S4, we report Wigner function measurements on states prepared by the qcMAP and ECD protocols in an earlier experimental setup. In this setup, we had $\chi_{\mathrm{qc}}/2\pi = 1.272$ MHz, $K_{\mathrm{c}}/2\pi = 2.33$ kHz, $\chi'_{\mathrm{qc}}/2\pi = 7.1$ kHz, cavity lifetime $T_{1,\mathrm{c}} = 128$ µs, qubit lifetime $T_1 = 6.3$ µs, and qubit coherence time $T_2^* = 2.4$ µs. In these experimental runs, the heat bath used to prepare the initial state was kept connected throughout the preparation and measurement protocol. Note that, in our setup, the cavity lifetime is limited by the external coupling via the coupling pin to the environment. Thus, the cavity coupling rate to the heat bath is the same as the cavity photon loss rate. The experiment was run with $\alpha = 2.5$ and $n_{\mathrm{th}} = 2.07$.

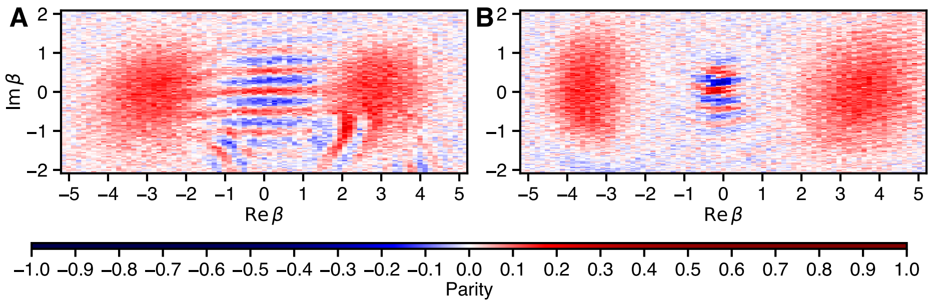

Fig. S3. Additional hot cat state measurements. (**A**) ECD protocol (**B**) qcMAP protocol. Note that this plot uses a linear scaling of the color bar. Here, the initial mean thermal cavity photon number is $n_{\mathrm{th}} = 1.84(3)$.

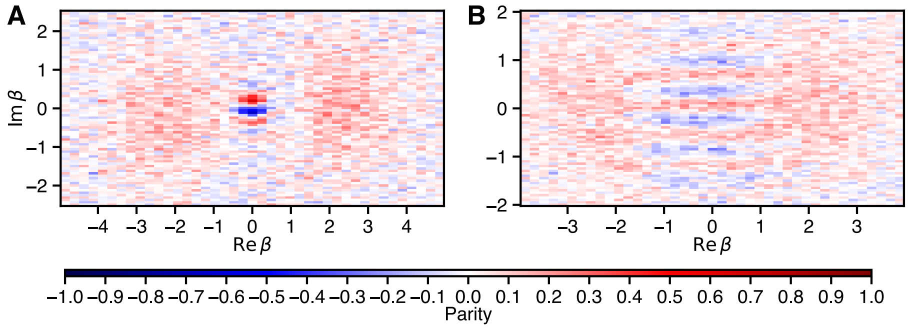

Fig. S4. Hot cat state measurements from a previous experimental setup with the heat bath left connected. (**A**) qcMAP protocol (**B**) ECD protocol. Note that this plot uses a linear scaling of the color bar. See section S1.3 for the parameters of this setup.

S1.4 Characterizing the Initial Thermal State

To determine the initial thermal state of the cavity mode, number-split qubit spectroscopy was performed (Figure S5). This allowed for the measurement of the cavity photon distribution. The steps involved are shown in Figure S5B. First, the cavity mode was equilibrated with the thermal bath as described in Section S1.1. Next, a cavity photon number selective qubit π-pulse was applied and the qubit state was measured. By repeating the measurement to get an ensemble average, the probability to excite the qubit at a certain frequency was determined.

Due to the cavity photon distribution and the dispersive coupling between the cavity and the qubit, the qubit resonance frequency is split into a spectrum of multiple frequencies where the relative resonance peak height depends on the cavity photon distribution (Figure S5C). By measuring the probability to excite the qubit across the frequency spectrum, we also directly measure the cavity photon number distribution.

For a thermal state with average photon occupation number n_{th}, the probability of measuring n photons is

$$P_{n_{\mathrm{th}}}(n) = \frac{n_{\mathrm{th}}^n}{(1+n_{\mathrm{th}})^{n+1}}. \tag{S1}$$

By fitting this to the spectral qubit excitation probability (Figure S5C), we determined n_{th} of the cavity state. By varying the added photon noise power, we found a relationship between the attenuator setting and n_{th} (Figure S5D).

The added thermal noise power is calculated from $P = k_B T f_{\mathrm{BW}}$. f_{BW} is the bandwidth of the cavity which is calculated from the cavity lifetime. T is the temperature of the resistor which is at room temperature and k_B is the Boltzman constant. The noise power is then reduced by the digital attenuator value. Critically, it is the measured mean thermal photon number or the cavity mode temperature that is used in the experiments and Fig. S5D just serves as a visual guide.

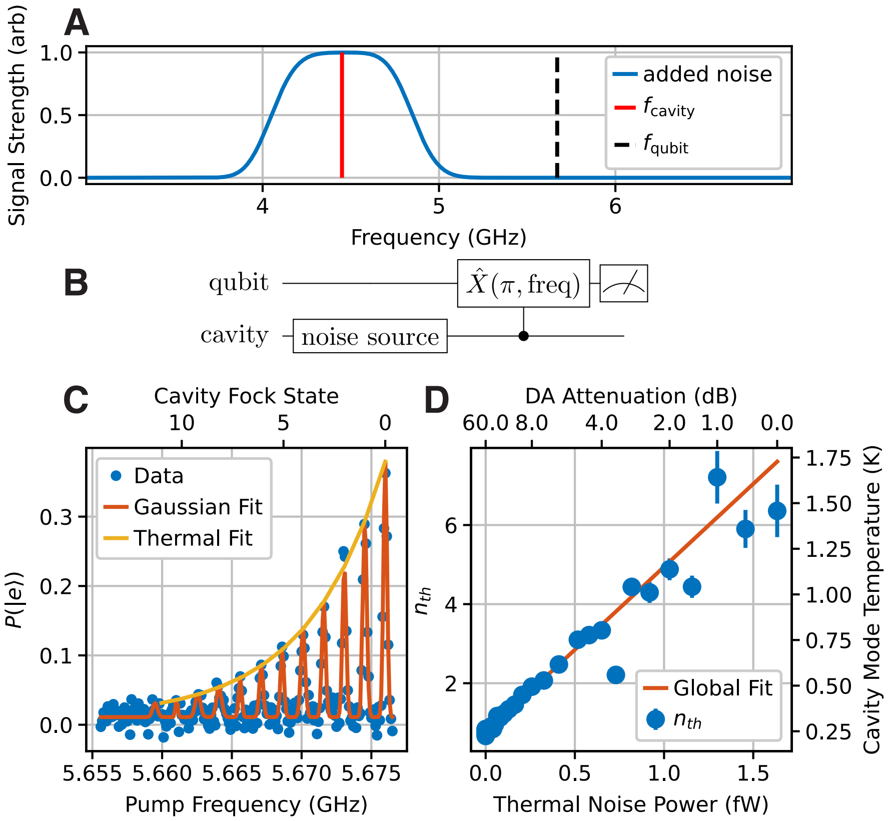

Fig. S5. Thermal State Measurement Technique (**A**) An illustration of frequency spectrum of the added noise. The thermal noise is only added at the cavity frequency while qubit frequencies are filtered out. The noise level is controlled by a digital attenuator. (**B**) Experimental Pulse sequence. (**C**) Qubit spectroscopy measurement result for a thermal state. Here, $n_{\text{th}} = 3.3(1)$. (**D**) Thermal population measurements for different attenuation settings, corresponding to noise powers. The added noise power with the mean thermal photon is fitted with a straight line.

S1.5 Characterization of Hamiltonian

The full system Hamiltonian, including perturbative terms due to higher excitation levels of the qubit and cavity nonlinearities, is

$$\hat{H}/\hbar = \omega_c \hat{c}^\dagger \hat{c} - \frac{K_c}{2}\hat{c}^\dagger\hat{c}^\dagger\hat{c}\hat{c} + \omega_q \hat{q}^\dagger\hat{q} - \frac{K_q}{2}\hat{q}^\dagger\hat{q}^\dagger\hat{q}\hat{q} - \chi_{qc}\hat{c}^\dagger\hat{c}\hat{q}^\dagger\hat{q} - \frac{K_c'}{6}\hat{c}^\dagger\hat{c}^\dagger\hat{c}^\dagger\hat{c}\hat{c}\hat{c} - \frac{\chi_{qc}'}{2}\hat{c}^\dagger\hat{c}^\dagger\hat{c}\hat{c}\hat{q}^\dagger\hat{q} \quad \text{(S2)}$$

Here $\hat{q}^\dagger$ and $\hat{c}^\dagger$ are the creation operators for the qubit and cavity mode respectively, and $\hat{q}, \hat{c}$ are the corresponding annihilation operators. The values of the Hamiltonian parameters were measured experimentally and are reported in Table S1.

To characterize the Hamiltonian of our system, we employed a measurement method to accurately determine the cavity frequency as a function of the cavity photon number and qubit initial state (Figure S6A). A similar technique was reported in *(11)*. First, the cavity was displaced by β and allowed to evolve for a time delay t, with the qubit in the ground state. The delay time was varied up to a maximum delay time, T. Subsequently, a second displacement with displacement parameter $\beta\exp\{-i\phi(t)\}$ was applied, where $\phi(t) = 2\pi \times 5t/T$. Finally, a cavity ground state selective π-pulse (described by the operator $\hat{X}(\pi, \sigma_t)$ with $\sigma_t = 300$ ns) was applied to the qubit, and the qubit state was measured.

The principle of the measurement is illustrated in Figure S6B. For weak Kerr effects and the qubit in the ground state, the displaced state approximately evolves as $|\beta e^{i\omega(\beta)t}\rangle$, where

$$\omega(\beta) = \Delta - |\beta|^2\frac{K_c}{2} - |\beta|^4\frac{K_c'}{6}, \quad \text{(S3)}$$

and the cavity frequency detuning $\Delta = \omega_c - \omega_{\text{drive}}$ from the drive frequency ω_{drive}. The ground-state selective π-pulse will flip the qubit only if $\phi(t)$ in the second displacement pulse matches $\omega(\beta)$, i.e. $\phi(t) = \omega(\beta)$. Thus, the probability of measuring the cavity in the ground state, or equivalently, qubit in the excited state, is expected to be

$$P(|e\rangle) = |\langle 0|\beta(t)\rangle|^2 = e^{-2|\beta|^2[1-\cos(\omega(\beta)t)] - t/T_{1,c}}, \quad \text{(S4)}$$

The exponential decay comes from the finite cavity lifetime.

We measure $P(|e\rangle)$ as a function of t and β (Figure S6C). We then fit Eq. (S4) as a function of t for the different values of β used in the measurement (Figure S6D). From the fit, we extract $\omega(\beta)$ for the given value of β. We then fit Eq. (S3) to the measured $\omega(\beta)$ to extract Δ, K_c, and K_c' (Figure S6E). Finally, we repeat the procedure with the qubit initially in the excited state, which allows us to determine χ_{qc} and χ_{qc}'.

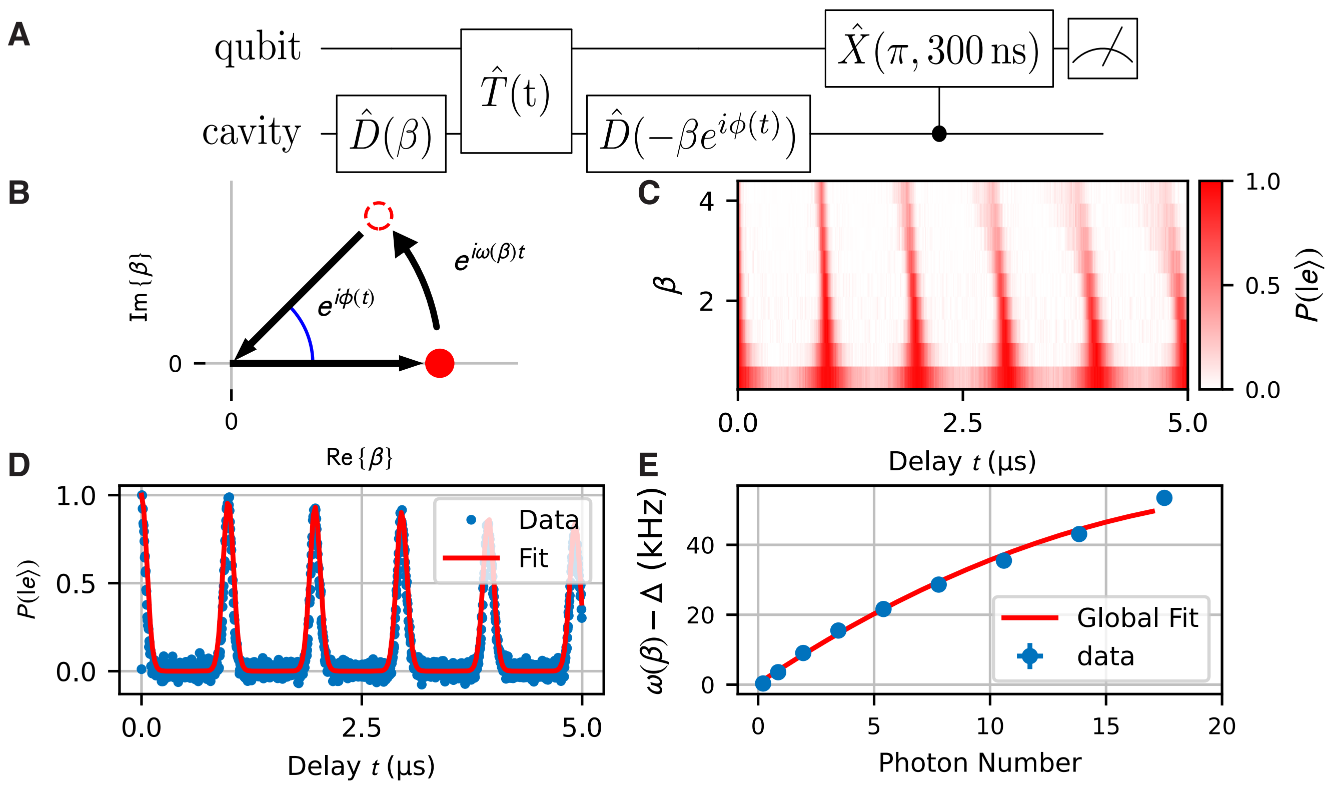

Fig. S6. Hamiltonian Measurement Technique (**A**) Experimental pulse sequence. (**B**) Phase space evolution of the cavity during the experiment. (**C**) Measurement data of the qubit excited state probability for different values of the delay time and the initial displacement. Here, the qubit is initialized in the ground state. (**D**) Measurement of the qubit excited state probability for a fixed value of β. The solid line is a fit of Eq. (S4). (**E**) The photon-number dependent cavity frequency $\omega(\beta)$ as a function of the initial average photon number $|\beta|^2$ with a fit of Eq. (S3).

Parameter	Symbol	Value
Qubit Frequency	$\omega_{\rm q}/2\pi$	5.676001 GHz
Qubit Anharmonicity	$K_{\rm q}/2\pi$	189.9 ± 0.4 MHz
Qubit Lifetime	T_1	31.0 ± 0.4 µs
Qubit Coherence Time	T_2^*	12.5 ± 0.4 µs
Qubit Hann Echo Time	T_2^E	16.9 ± 0.5 µs
High Q Cavity Frequency	$\omega_{\rm c}/2\pi$	4.544939 GHz
High Q Cavity Self-Kerr	$K_{\rm c}/2\pi$	4.9 ± 0.1 kHz
High Q Cavity Second Order Self-Kerr	$K'_{\rm c}/2\pi$	14 ± 8 Hz
High Q Cavity Lifetime	$T_{1,\rm c}$	110 ± 2 µs
High Q Cavity - Qubit Dispersive Shift	$\chi_{\rm qc}/2\pi$	1.499 ± 0.003 MHz
High Q Cavity - Qubit Second Order Dispersive shift	$\chi'_{\rm qc}/2\pi$	12.8 ± 0.9 kHz
High Q Cavity Residual Mean Thermal Photon Number	$n_{\rm th,\ residual}$	0.0338 ± 0.007
High Q Cavity Residual Mode Temperature	$T_{\rm cavity,\ residual}$	63.7 ± 0.4 mK
Qubit Residual Mode Temperature	$T_{\rm qubit}$	68 ± 1 mK
Readout Resonator Frequency	$\omega_{\rm r}/2\pi$	7.528852 GHz
Readout Resonator External Coupling	$\kappa_{\rm c\ ext}/2\pi$	1.33 MHz
Readout Resonator - Qubit Dispersive Shift	$\chi_{\rm rq}/2\pi$	1.61 MHz
Readout Resonator - High Q Cavity Dispersive Shift (calculated)	$\chi_{\rm rc,cal}/2\pi$	3.4 kHz

Table. S1. Hamiltonian Parameters. The high Q cavity lifetime is limited by its external coupling. The reported cross Kerr $\chi_{\rm readout-cavity,cal}$ is a lower bound calculated via $\chi_{\rm rc} = \chi_{\rm qc}\chi_{\rm qr}(\frac{1}{\Delta_{\rm qc}} + \frac{1}{\Delta_{\rm qr}})$.

S1.6 Density matrix reconstruction

In general, the density matrix $\hat{\rho}$ can be obtained from the Wigner function $W(\beta)$ as

$$\hat{\rho} = 2\int \mathrm{d}^2\beta\, W(\beta)\hat{\Pi}(\beta). \tag{S5}$$

This equation is the inverse of $W(\beta) = \frac{2}{\pi}\mathrm{Tr}\left\{\hat{\rho}\hat{\Pi}(\beta)\right\}$ and can be shown by writing out $\hat{\Pi}(\beta)$ in a known basis, e.g. $\hat{\Pi}(\beta) = \int_{-\infty}^{\infty} \mathrm{d}x\, \mathrm{e}^{-2\mathrm{i}x\sqrt{2}\mathrm{Im}\{\beta\}}|\sqrt{2}\mathrm{Re}\{\beta\} - x\rangle\langle\sqrt{2}\mathrm{Re}\{\beta\} + x|$ where $|x\rangle$ is an eigenket of $\hat{x} = (\hat{c} + \hat{c}^\dagger)/\sqrt{2}$ (*16*). To reconstruct the density matrices of the states prepared, we choose to compute the matrix elements in the Fock basis

$$\rho_{mn} = \langle m|\hat{\rho}|n\rangle = 2\int \mathrm{d}^2\beta\, W(\beta)\langle m|\hat{\Pi}(\beta)|n\rangle \tag{S6}$$

using our measured data for $W(\beta)$. The operator $\hat{\Pi}(\beta)$ has Fock basis matrix elements (*20*)

$$\langle m|\hat{\Pi}(\beta)|n\rangle = (-1)^n \cdot \begin{cases} \sqrt{\dfrac{n!}{m!}}(2\beta)^{m-n}\mathrm{e}^{-2|\beta|^2}\mathcal{L}_n^{(m-n)}(4|\beta|^2) & (m \geq n) \\ \sqrt{\dfrac{m!}{n!}}(-2\beta^*)^{n-m}\mathrm{e}^{-2|\beta|^2}\mathcal{L}_m^{(n-m)}(4|\beta|^2) & (n \geq m) \end{cases} \tag{S7}$$

where $\mathcal{L}_n^{(k)}(x)$ are the associated Laguerre polynomials. The Wigner function data is given as averages on a grid β_{rj} corresponding to experimentally measured values of β, with N_R, N_I the dimensions of the grid and $\Delta_{R,I}$ the grid spacing along the real and imaginary directions. The measured Wigner function data is the $N_R \times N_I$ matrix $W_{rj} = W(\beta_{rj})$. We can then discretize Eq. (S6) on β_{rj} to find

$$\rho_{mn} \approx 2\Delta_R\Delta_I \sum_{r=0}^{N_R-1}\sum_{j=0}^{N_I-1} W_{rj}\Pi_{mn,rj} \tag{S8}$$

which is approximate due to the finite size of the grid, and where we defined

$$\Pi_{mn,rj} = \langle m|\hat{\Pi}(\beta_{rj})|n\rangle. \tag{S9}$$

With this method, we can estimate the density matrix directly from the data. Recall that a proper density matrix must have unit trace $\mathrm{Tr}\,\hat{\rho} = 1$, be hermitian $\hat{\rho}^\dagger = \hat{\rho}$, and be positive semidefinite. The estimate ρ_{mn} obtained from Eq. (S8) will be hermitian, but not strictly normalized nor positive semidefinite. This is for three reasons: 1) The measurements contain additive randomly distributed noise which propagates into the matrix ρ_{mn}. 2) The data grid has a finite size, which makes Eq. (S8) be an inexact approximation to Eq. (S5), in particular for large m and n. 3) As explained in Section S4.1, the Wigner measurement does not strictly produce a Wigner function but rather the function

$$W_{\mathrm{meas.}}(\beta) = p_g W_g(\beta) - p_e W_e(\beta) \tag{S10}$$

where $p_{g,e}$ are the qubit $|g\rangle$ and $|e\rangle$ populations at the end of the state preparation, and $W_{g,e}(\beta)$ are respectively the Wigner functions corresponding to the reduced density matrices $\hat{\rho}_g = \langle g|\hat{\rho}_{\rm tot}|g\rangle$ and $\hat{\rho}_e = \langle e|\hat{\rho}_{\rm tot}|e\rangle$ of the total oscillator-qubit density matrix $\hat{\rho}_{\rm tot.}$. Due to the linearity of Eq. (S5), our density matrix reconstruction therefore gives us the matrix elements

$$\mu_{mn} = \langle m|p_g\hat{\rho}_g - p_e\hat{\rho}_e|n\rangle. \tag{S11}$$

As $\hat{\rho}_e$ is positive semidefinite and $p_e \geq 0$, the term $-p_e\hat{\rho}_e$ is negative semidefinite and thus makes the matrix μ indefinite under the conditions of our experiment. Out of the three error sources, we find 3) to be the most important in our case. We are seeking to estimate the density matrix $\hat{\rho}_g$, with $\hat{\rho}_e$ a perturbation. Efficient max-likelihood estimators for the density matrix such as (*44*) are not designed for this scenario. We therefore choose to obtain our final estimate for ρ_{mn} by truncating all negative eigenvalues of μ. More specifically, we diagonalize $\mu = UDU^\dagger$, where D is the diagonal matrix of eigenvalues and U the matrix of eigenvectors of μ, and then construct the matrix $\rho = U\max(D,0)U^\dagger/\mathrm{Tr}\left\{\max(D,0)\right\}$, where the max operation applies elementwise. This provides our final estimate ρ_{mn}, which we plot in Figure 1H-J in the main text.

Using ρ_{mn} as the reconstructed density matrix of the prepared state, we can compute its representation in the eigenbasis of the position operator $\hat{x} = (\hat{a}+\hat{a}^\dagger)/\sqrt{2}$, and then its coherence function. The position representation density matrix can be computed as

$$\rho(x_1,x_2) = \sum_l p_l[U\boldsymbol{\psi}(x_1)]_l[U\boldsymbol{\psi}(x_2)]_l^* \tag{S12}$$

where p_l are the eigenvalues of ρ_{mn}, $\boldsymbol{\psi}(x)$ is a vector with elements $\langle x|n\rangle = \mathrm{e}^{-x^2/2}H_n(x)/\sqrt{2^n n!\sqrt{\pi}}$ where $H_n(x)$ are the Hermite polynomials, and U is the eigenvector matrix of ρ_{mn}. Using this expression for $\rho(x_1,x_2)$, we compute the coherence function as

$$g(x_1,x_2) = \frac{|\rho(x_1,x_2)|}{\sqrt{\rho(x_1,x_1)\rho(x_2,x_2)}}. \tag{S13}$$

Using this method, we obtain the coherence function estimates plotted in Figure 4 in the main text.

S1.7 Hot cat state fringe lifetime measurement

We measure the decay of the fringes of the hot cat states by measuring the parity of the cat state at the phase space location (0,0) after a delay time.

The fringe lifetime is related to the cavity lifetime by (*45*)

$$T_{\text{fringe}} = \frac{T_{1,\text{c}}}{2|\alpha|^2(2n_{\text{b}}+1)}, \tag{S14}$$

where α is the cat size and n_{b} is the mean thermal excitation number of the bath the system is connected to (note that this is not the initial mean thermal photon population). Using the values in Table S1, we estimate the expected fringe lifetime to be 4.7 ± 0.9 µs.

Figure S7 shows the measurement of the fringe lifetime for hot cat states. The average lifetime across all hot cat states is 3.7 ± 0.2 µs. The discrepancy with the expected lifetime indicates the presence of decoherence channels beyond those characterised in Table S1.

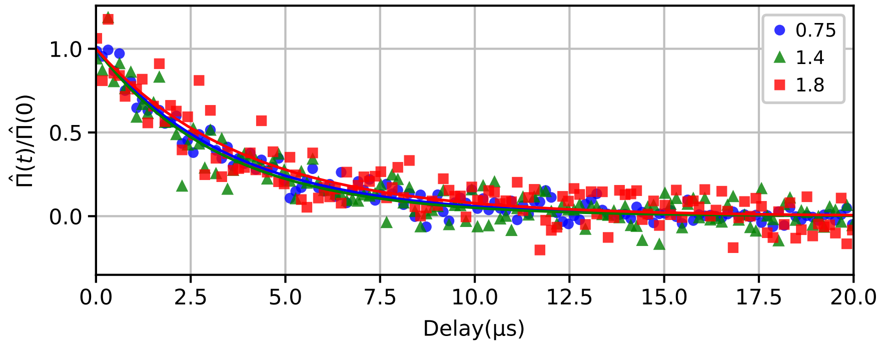

Fig. S7. Hot cat state fringe lifetime. The label refers to the initial thermal excitation number in the cavity $n_{\rm th}$. The y-values are normalized to the initial parity of the fringe at zero delay. The lines are exponential fits from which the average lifetime across all hot cat states is obtained to be 3.7 ± 0.2 μs.

S2 Theory

We show that the qcMAP and ECD protocols are described by the operators $\hat{S}_{1,2}$ under ideal conditions, derive the hot cat Wigner functions $W_{1,2}(\beta)$, and analyze the coherence functions of the hot cats.

S2.1 Theoretical analysis of the qcMAP and ECD protocols

In this section, we show that the ECD and qcMAP pulse sequences displayed in the quantum circuit diagrams in Figure 2A-C are respectively equivalent to applying the operators $\hat{S}_1'$ and $\hat{S}_2$ to the initial cavity state $\hat{\rho}_0$. A necessary condition is that $\hat{\rho}_0$ has no overlap with itself when displaced by 2α. We repeat the operator definitions from the main text for convenience. The equivalent operator for the ECD protocol is

$$\hat{S}_1' \equiv \frac{1}{\sqrt{2}} \left[\hat{D}(\alpha) - \mathrm{e}^{\mathrm{i}(\phi+2|\alpha|^2)} \hat{D}(-\alpha) \right] \mathrm{i}^{\hat{n}}, \tag{S15}$$

which in turn is equivalent to the operator

$$\hat{S}_1 \equiv \frac{1}{\sqrt{2}} \left[\hat{D}(\alpha) + \mathrm{e}^{\mathrm{i}\phi} \hat{D}(-\alpha) \right] \tag{S16}$$

when the initial state is thermal and ϕ is experimentally controllable. The equivalent operator for the qcMAP protocol is

$$\hat{S}_2 \equiv \frac{1}{\sqrt{2}} \left[1 - \mathrm{e}^{\mathrm{i}\phi} \hat{\Pi} \right] \hat{D}(\alpha). \tag{S17}$$

From Figure 2A-C, we read that the ECD and qcMAP protocols first prepare the initial thermal state $\hat{\rho}_0$ of the cavity and then apply the unitary operators

$$\hat{U}_1 \equiv \hat{D}(\alpha)\hat{X}(\pi,\sigma_t)\hat{D}(-\alpha)\hat{T}(\pi/2\chi_{\mathrm{qc}})\hat{D}[-\alpha\tfrac{(1+\mathrm{i})}{2}]\hat{Y}(\pi)\hat{D}[-\alpha\tfrac{(1+\mathrm{i})}{2}]\hat{T}(\pi/2\chi_{\mathrm{qc}})\hat{D}(\alpha)\hat{X}(\pi/2,\phi) \tag{S18}$$

for ECD, and

$$\hat{U}_2 \equiv \hat{D}(-\alpha)\hat{X}(\pi,\sigma_t)\hat{D}(\alpha)\hat{T}(\pi/\chi_{\mathrm{qc}})\hat{D}(\alpha)\hat{X}(\pi/2,\phi) \tag{S19}$$

for qcMAP, to the cavity-qubit initial state $\hat{\rho}_0|g\rangle\langle g|$ (we define all operators appearing in these expressions in the next paragraph). Under ideal conditions, the final state of the protocols is

$$\hat{U}_i\hat{\rho}_0|g\rangle\langle g|\hat{U}_i^\dagger = \hat{\rho}_i|g\rangle\langle g| \tag{S20}$$

($i \in \{1,2\}$), with

$$\hat{\rho}_i \equiv \hat{S}_{gg,i}\hat{\rho}_0\hat{S}_{gg,i}^\dagger, \tag{S21}$$

$$\hat{S}_{gg,i} \equiv \langle g|\hat{U}_i|g\rangle. \tag{S22}$$

It is straightforward to show that the final cavity-qubit state Eq. (S20) is a product state, with the qubit in the $|g\rangle$ state, if and only if

$$\mathrm{Tr}\,\{\hat{S}_{gg,i}\hat{\rho}_0\hat{S}^\dagger_{gg,i}\} = 1. \tag{S23}$$

Consequently, the ECD and qcMAP protocols, given exactly by $\hat{U}_{1,2}$, are equivalent to the operators $\hat{S}_{gg,\{1,2\}}$ for all initial states $\hat{\rho}_0$ which satisfy Eq. (S23).

The operators appearing in Eqs. (S18) and (S19) are: I) The cavity displacement operator

$$\hat{D}(\beta) \equiv \exp\left\{\beta\hat{c}^\dagger - \beta^*\hat{c}\right\} \tag{S24}$$

where $\hat{c}, \hat{c}^\dagger$ are the cavity annihilation and creation operators and β is a complex-valued argument. II) The time-evolution operator under the dispersive interaction Hamiltonian $\hat{T}(t)$. The interaction Hamiltonian is $\hat{H} = -\hbar\chi_{\mathrm{qc}}\hat{n}|e\rangle\langle e|$, where $\hat{n} = \hat{c}^\dagger\hat{c}$, so the time-evolution operator is

$$\hat{T}(t) = \exp\{\mathrm{i}t\chi_{\mathrm{qc}}\hat{n}|e\rangle\langle e|\} = |g\rangle\langle g| + \exp\{\mathrm{i}t\chi_{\mathrm{qc}}\hat{n}\}|e\rangle\langle e|. \tag{S25}$$

In particular,

$$\hat{T}(\pi/\chi_{\mathrm{qc}}) = |g\rangle\langle g| + \hat{\Pi}|e\rangle\langle e| \tag{S26}$$

where $\hat{\Pi} = (-1)^{\hat{n}}$ is the parity operator, and

$$\hat{T}(\pi/2\chi_{\mathrm{qc}}) = |g\rangle\langle g| + \mathrm{i}^{\hat{n}}|e\rangle\langle e|. \tag{S27}$$

In phase space, the operator $\mathrm{i}^{\hat{n}}$ leads to a counterclockwise rotation by $\pi/2$ around the phase-space origin. III) The qubit rotation operators acting only on the qubit Hilbert space

$$\hat{X}(\pi/2,\phi) \equiv \frac{1}{\sqrt{2}}\left[\left(|g\rangle + \mathrm{i}e^{\mathrm{i}\phi}|e\rangle\right)\langle g| + \left(|e\rangle + \mathrm{i}e^{-\mathrm{i}\phi}|g\rangle\right)\langle e|\right], \tag{S28}$$

where ϕ is an experimentally controllable phase,

$$\hat{X}(\pi) \equiv \mathrm{i}\left(|g\rangle\langle e| + |e\rangle\langle g|\right), \tag{S29}$$

and

$$\hat{Y}(\pi) \equiv \hat{R}(\pi,\pi/2) = |g\rangle\langle e| - |e\rangle\langle g|. \tag{S30}$$

These operators are special cases of the general qubit rotation operator

$$\hat{R}(a,\boldsymbol{u}) \equiv \exp\left\{\frac{\mathrm{i}a}{2}\boldsymbol{u}\cdot\hat{\boldsymbol{\sigma}}\right\} = \hat{1}\cos\frac{a}{2} + \mathrm{i}\boldsymbol{u}\cdot\hat{\boldsymbol{\sigma}}\sin\frac{a}{2} \tag{S31}$$

with a a real variable, $\boldsymbol{u}$ a unit vector on the 3-dimensional unit sphere, and $\hat{\boldsymbol{\sigma}} = \hat{\sigma}_x\boldsymbol{e}_x + \hat{\sigma}_y\boldsymbol{e}_y + \hat{\sigma}_z\boldsymbol{e}_z$ ($\boldsymbol{e}_j$ are the coordinate unit vectors and $\hat{\sigma}_j$ the Pauli matrices $\hat{\sigma}_x = |g\rangle\langle e| + |e\rangle\langle g|$, $\hat{\sigma}_y = \mathrm{i}(-|g\rangle\langle e| + |e\rangle\langle g|)$, $\hat{\sigma}_z = |g\rangle\langle g| - |e\rangle\langle e|$). We have $\hat{X}\,(\pi/2,\phi) = \hat{R}(\pi/2, \cos\phi\;\boldsymbol{e}_x + \sin\phi\;\boldsymbol{e}_y)$,

$\hat{X}(\pi) = \hat{R}(\pi, \boldsymbol{e}_x)$, $\hat{Y}(\pi) = \hat{R}(\pi, \boldsymbol{e}_y)$. IV) The cavity-selective qubit rotation operator $\hat{X}(\pi, \sigma_t)$. We assume that it takes the form

$$\hat{X}(\pi, \sigma_t) = \sum_{n=0}^{\infty} |n\rangle\langle n| \hat{R}(a_n, \boldsymbol{u}_n), \tag{S32}$$

i.e. that it is defined by specifying a sequence of qubit rotation operators on the Fock states $|n\rangle$. We further assume that the parameters $\{a_n\}_{n=0}^{\infty}$ and $\{\boldsymbol{u}_n\}_{n=0}^{\infty}$ are such that for $n \leq N$, where N is a number that depends on σ_t, $a_n = \pi$ and $\boldsymbol{u}_n = \boldsymbol{e}_x$. Additionally, the sequence of parameters $\{a_n\}_{n=0}^{\infty}$ decays so that there is a number M (also determined by σ_t) such that $a_n = 0$ when $n > M$. In this case, we can write

$$\hat{X}(\pi, \sigma_t) = \mathrm{i}\hat{P}_{\leq N}\hat{\sigma}_x + \hat{P}_{>M} + \hat{Q}_{NM}, \tag{S33}$$

where we have introduced the operators

$$\hat{P}_{\leq N} \equiv \sum_{n=0}^{N} |n\rangle\langle n|, \tag{S34}$$

$$\hat{P}_{>M} \equiv \sum_{n=M+1}^{\infty} |n\rangle\langle n|, \tag{S35}$$

$$\hat{Q}_{NM} \equiv \sum_{n=N+1}^{M} |n\rangle\langle n| \hat{R}(a_n, \boldsymbol{u}_n). \tag{S36}$$

It is possible (by using a Magnus approximation (*18*)) to find an explicit expression for the operator $\hat{X}(\pi, \sigma_t)$ that results when a Gaussian qubit pulse is applied to our dispersively coupled cavity-qubit system. That expression is indeed well-described by Eq. (S33). However, for the present discussion, we do not need to specify $\hat{X}(\pi, \sigma_t)$ beyond the description already made.

We now show the equivalence of $\hat{U}_{1,2}$ to $\hat{S}_{1,2}$ under conditions which we identify during the analysis. We begin with the qcMAP protocol $\hat{U}_2$. From the definitions made, $\hat{U}_2$ can be rewritten as

$$\hat{U}_2 = \frac{1}{\sqrt{2}}\hat{D}(-\alpha)\hat{X}(\pi, \sigma_t)\left[\hat{D}(2\alpha)|g\rangle\left(\langle g| + \mathrm{i}e^{\mathrm{i}\phi}\langle e|\right) + \hat{\Pi}|e\rangle\left(\mathrm{i}e^{\mathrm{i}\phi}\langle g| + \langle e|\right)\right] \tag{S37}$$

from which we identify

$$\begin{aligned} \hat{S}_{gg,2} &= \frac{1}{\sqrt{2}}\hat{D}(-\alpha)\left[\langle g|\hat{X}(\pi, \sigma_t)|g\rangle\hat{D}(2\alpha) + \mathrm{i}e^{\mathrm{i}\phi}\hat{\Pi}\langle g|\hat{X}(\pi, \sigma_t)|e\rangle\right] \\ &= \frac{1}{\sqrt{2}}\hat{D}(-\alpha)\left[\left(\hat{P}_{>M} + \langle g|\hat{Q}_{NM}|g\rangle\right)\hat{D}(2\alpha) + \mathrm{i}e^{\mathrm{i}\phi}\hat{\Pi}\left(\mathrm{i}\hat{P}_{\leq N} + \langle g|\hat{Q}_{NM}|e\rangle\right)\right] \end{aligned} \tag{S38}$$

where we inserted Eq. (S33) to go from the first to the second line. We now wish to identify the initial states $\hat{\rho}_0$ for which $\hat{S}_{gg,2}$ fulfils Eq. (S23). Consider first an initial state $\hat{\rho}_0$ which has non-zero matrix elements only in the first N Fock states, i.e.

$$\hat{\rho}_0 = \sum_{k,l=0}^{N} \langle k|\hat{\rho}_0|l\rangle |k\rangle\langle l|. \tag{S39}$$

For this $\hat{\rho}_0$, $\hat{P}_{\leq N}\hat{\rho}_0 = \hat{\rho}_0$. If we have additionally chosen α large enough so that the matrix element $\langle M|\hat{D}(2\alpha)|M\rangle$ is negligible, then it also follows (using the triangle inequality) that $\hat{P}_{\leq M}\hat{D}(2\alpha)\hat{\rho}_0 = 0$. In a Wigner function picture, the condition on α can be formulated as the M:th Fock state Wigner function being negligible for arguments larger than $|\alpha|$, i.e. $W_{|M\rangle}(|\beta| \geq |\alpha|) = 0$. From $\hat{P}_{\leq N}\hat{\rho}_0 = \hat{\rho}_0$ and $\hat{P}_{\leq M}\hat{D}(2\alpha)\hat{\rho}_0 = 0$ it follows that $\hat{Q}_{NM}\hat{\rho}_0 = 0$, $\hat{Q}_{NM}\hat{D}(2\alpha)\hat{\rho}_0 = 0$, and $\hat{P}_{>M}\hat{D}(2\alpha)\hat{\rho}_0 = (1 - \hat{P}_{\leq M})\hat{D}(2\alpha)\hat{\rho}_0 = \hat{D}(2\alpha)\hat{\rho}_0$. We therefore obtain

$$\hat{S}_{gg,2}\hat{\rho}_0 = \frac{1}{\sqrt{2}}\left[\hat{D}(\alpha) - \mathrm{e}^{\mathrm{i}\phi}\hat{D}(-\alpha)\hat{\Pi}\right]\hat{\rho}_0 = \hat{S}_2\hat{\rho}_0. \tag{S40}$$

The condition Eq. (S23) is also satisfied. Consequently, $\hat{U}_2\hat{\rho}_0|g\rangle\langle g|\hat{U}_2^\dagger = \hat{S}_2\hat{\rho}_0\hat{S}_2^\dagger|g\rangle\langle g|$, and $\hat{S}_2$ accurately describes the action of the qcMAP protocol, for all $\hat{\rho}_0$ of the form Eq. (S39) when α is such that $W_{|M\rangle}(|\beta| \geq |\alpha|) = 0$.

Identical arguments show the equivalence of $\hat{U}_1$ to $\hat{S}_1'$. From Eq. (S18), $\hat{U}_1$ can be written

$$\hat{U}_1 = \frac{1}{\sqrt{2}}\hat{D}(\alpha)\hat{X}(\pi,\sigma_t)\left[\hat{D}(-2\alpha)\mathrm{e}^{\mathrm{i}|\alpha|^2}|g\rangle\left(\langle g|\mathrm{i}\mathrm{e}^{\mathrm{i}\phi} + \langle e|\right) - \mathrm{e}^{-\mathrm{i}|\alpha|^2}|e\rangle\left(\langle g| + \mathrm{i}\mathrm{e}^{-\mathrm{i}\phi}\langle e|\right)\right]\mathrm{i}^{\hat{n}}. \tag{S41}$$

We identify

$$\hat{S}_{gg,1} = \frac{1}{\sqrt{2}}\hat{D}(\alpha)\left[\mathrm{i}\mathrm{e}^{\mathrm{i}\phi}\left(\hat{P}_{>M} + \langle g|\hat{Q}_{NM}|g\rangle\right)\hat{D}(-2\alpha)\mathrm{e}^{\mathrm{i}|\alpha|^2} - \mathrm{e}^{-\mathrm{i}|\alpha|^2}\left(\mathrm{i}\hat{P}_{\leq N} + \langle g|\hat{Q}_{NM}|e\rangle\right)\right]\mathrm{i}^{\hat{n}}. \tag{S42}$$

When this operator acts on a state $\hat{\rho}_0$ described by Eq. (S39), and α is large enough so that $\hat{P}_{\leq M}\hat{D}(2\alpha)\hat{\rho}_0 = 0$ as before, then

$$\hat{S}_{gg,1}\hat{\rho}_0 = \frac{-\mathrm{i}\mathrm{e}^{-\mathrm{i}|\alpha|^2}}{\sqrt{2}}\left[\hat{D}(\alpha) - \mathrm{e}^{\mathrm{i}(\phi+2|\alpha|^2)}\hat{D}(-\alpha)\right]\mathrm{i}^{\hat{n}}\hat{\rho}_0 = -\mathrm{i}\mathrm{e}^{-\mathrm{i}|\alpha|^2}\hat{S}_1'\hat{\rho}_0. \tag{S43}$$

Eq. (S23) is satisfied. The global phase vanishes when considering $\hat{S}_{gg,1}\hat{\rho}_0\hat{S}_{gg,1}^\dagger$.

We now relax the assumption that $\hat{\rho}_0$ is of the form Eq. (S39). In general, the state after the ECD and qcMAP protocols can be written

$$\hat{U}_i\hat{\rho}_0|g\rangle\langle g|\hat{U}_i^\dagger = p_g\hat{\rho}_g|g\rangle\langle g| + p_e\hat{\rho}_e|e\rangle\langle e| + \hat{\psi} \tag{S44}$$

where

$$p_g = \mathrm{Tr}\,\{\hat{S}_{gg,i}\hat{\rho}_0\hat{S}^\dagger_{gg,i}\}, \tag{S45}$$

$$p_e = 1 - p_g, \tag{S46}$$

are the probabilities of finding the qubit in the $|g\rangle$ and $|e\rangle$ states,

$$\hat{\rho}_g \equiv \frac{\hat{S}_{gg,i}\hat{\rho}_0\hat{S}^\dagger_{gg,i}}{p_g}, \tag{S47}$$

$$\hat{\rho}_e \equiv \frac{\hat{S}_{eg,i}\hat{\rho}_0\hat{S}^\dagger_{eg,i}}{p_e} \tag{S48}$$

are the qubit-conditional cavity states (where $\hat{S}_{eg,i} \equiv \langle e|\hat{U}_i|g\rangle$), and $\hat{\psi}$ represents off-diagonal terms in the qubit basis. The previous condition Eq. (S23) is $p_g = 1$. For initial states $\hat{\rho}_0$ which have non-negligible matrix elements only for Fock numbers $\leq N$, i.e. are described by Eq. (S39) plus a negligible part, one has $p_g \approx 1$, $p_e \approx 0$. More precisely, one computes

$$p_g = \frac{1}{2}\left[\langle\hat{P}_{\leq N}\rangle + \langle\hat{D}(-2\alpha)\hat{P}_{>M}\hat{D}(2\alpha)\rangle + q(\sigma_t, \alpha, n_{\mathrm{th}})\right] \tag{S49}$$

where the expectation value is with respect to $\hat{\rho}_0$, and q represents terms related to expectation values of $\hat{Q}_{NM}$. If both the first two terms are 1, then $q(\sigma_t, \alpha, n_{\mathrm{th}}) = 0$. If $\langle\hat{P}_{\leq N}\rangle \approx 1$, it is always possible to choose α such that also $\langle\hat{D}(-2\alpha)\hat{P}_{>M}\hat{D}(2\alpha)\rangle \approx 1$ and therefore $q(\sigma_t, \alpha, n_{\mathrm{th}}) \approx 0$. For these states, the equivalence of $\hat{U}_{1,2}$ to $\hat{S}_{1,2}$ can therefore be satisfied to arbitrary precision in principle, and it is a good approximation to consider the ECD and qcMAP protocols equivalent to $\hat{S}_{1,2}$. The thermal state

$$\hat{\rho}_T \equiv \frac{1}{n_{\mathrm{th}}+1}\sum_{n=0}^{\infty}\left(\frac{n_{\mathrm{th}}}{n_{\mathrm{th}}+1}\right)^n |n\rangle\langle n|, \tag{S50}$$

is a particular example of a state which can be considered to have negligible representation for Fock numbers above N. It has

$$\mathrm{Tr}\,\{\hat{P}_{>N}\hat{\rho}_T\} = \left(\frac{n_{\mathrm{th}}}{n_{\mathrm{th}}+1}\right)^{N+1} \tag{S51}$$

which goes to 0 in the limit $N \to \infty$. In practice, the choice of σ_t (i.e. N and M) and α are restricted by experimental limitations, and the nonzero p_e leads to perturbations from the ideal result (i.e. that described by $\hat{S}_{1,2}$).

The conditions derived so far are those under which our protocols become equivalent to the operators $\hat{S}_{1,2}$. As a final remark, we note that one can also study the conditions for the operators

$\hat{S}_{1,2}$ to be effectively unitary independently of how they are implemented. This can be done by replacing $\hat{S}_{gg,i}$ with $\hat{S}_i$ in Eq. (S23). For a general initial state $\hat{\rho}_0$, one then has

$$\mathrm{Tr}\left\{\hat{S}_1\hat{\rho}_0\hat{S}_1^\dagger\right\} = 1 - \mathrm{Re}\left\{\mathrm{e}^{-\mathrm{i}\phi}\chi_0(2\alpha)\right\} = 1, \tag{S52}$$

$$\mathrm{Tr}\left\{\hat{S}_2\hat{\rho}_0\hat{S}_2^\dagger\right\} = 1 - \frac{\pi}{2}\cos\phi\, W_0(\alpha) = 1. \tag{S53}$$

Here, $W_0(\alpha) = 2\pi^{-1}\mathrm{Tr}\,\{\hat{\Pi}(\alpha)\hat{\rho}_0\}$ and $\chi_0(\alpha) = \mathrm{Tr}\,\{\hat{D}(\alpha)\hat{\rho}_0\}$ are the Wigner and characteristic functions of the initial state. These equations can be satisfied either by choice of ϕ or α. If we want this equation to be satisfied for any ϕ, this gives the necessary conditions that $\chi_0(|\beta| > 2|\alpha|) = 0$ ($\hat{S}_1$) and $W_0(|\beta| > |\alpha|) = 0$ ($\hat{S}_2$). We also see that if ϕ is a half-integer multiple of π, the operator $\hat{S}_2$ is unitary independently of α. For states that have a constant-phase characteristic function (e.g. thermal states), there are also choices of ϕ for which $\hat{S}_1$ is effectively unitary independently of α. To avoid confusion, we stress that ϕ does not enter any of the conditions for the ECD and qcaMAP protocols to be equivalent to the operators $\hat{S}_{1,2}$.

S2.2 Derivation of the Wigner functions $W_{1,2}(\beta)$ from the operators $\hat{S}_{1,2}$

We give derivations of the Wigner functions $W_{1,2}(\beta)$ that result when the operators $\hat{S}_{1,2}$ are applied to an initial state $\hat{\rho}_0$ and in particular the thermal state $\hat{\rho}_T$. Starting from

$$W_{1,2}(\beta) = \frac{2}{\pi}\mathrm{Tr}\,\{\hat{\Pi}(\beta)\hat{S}_{1,2}\hat{\rho}_0\hat{S}_{1,2}^\dagger\} \tag{S54}$$

where $\hat{\Pi}(\beta) = \hat{D}(\beta)\hat{\Pi}\hat{D}^\dagger(\beta)$, one uses the cyclic property of the trace to express $W_{1,2}(\beta)$ as the expectation value of the operator $\hat{S}_{1,2}^\dagger\hat{\Pi}(\beta)\hat{S}_{1,2}$ in the initial state. This allows us to express the Wigner functions $W_{1,2}(\beta)$ in terms of the Wigner function of the initial state $W_0(\beta)$, which is given by

$$W_0(\beta) = \frac{2}{\pi}\mathrm{Tr}\,\{\hat{\Pi}(\beta)\hat{\rho}_0\}. \tag{S55}$$

Here, $\hat{\rho}_0$ is any state for which the operators $\hat{S}_{1,2}$ accurately describe the outcome of the ECD and qcMAP protocols (see Section S2.1). One computes

$$\hat{S}_1^\dagger\hat{\Pi}(\beta)\hat{S}_1 = \frac{1}{2}\Big\{\hat{\Pi}\left(\beta-\alpha\right) + \hat{\Pi}\left(\beta+\alpha\right) - 2\cos\left(4\mathrm{Im}\left\{\alpha^*\beta\right\} + \phi\right)\hat{\Pi}(\beta)\Big\} \tag{S56}$$

and consequently

$$W_1(\beta) = \frac{1}{2}\Big\{W_0\left(\beta-\alpha\right) + W_0\left(\beta+\alpha\right) - 2\cos\left(4\mathrm{Im}\left\{\alpha^*\beta\right\} + \phi\right)W_0(\beta)\Big\}. \tag{S57}$$

If one instead of $\hat{S}_1$ uses $\hat{S}_1'$ (Eq. (S15)), the result is

$$W_{1'}(\beta) = \frac{1}{2}\Big\{W_0\left[-\mathrm{i}(\beta-\alpha)\right] + W_0\left[-\mathrm{i}(\beta+\alpha)\right] - 2\cos\left(4\mathrm{Im}\left\{\alpha^*\beta\right\} + \phi + 2|\alpha|^2\right)W_0(-\mathrm{i}\beta)\Big\}. \tag{S58}$$

Further, one computes

$$\hat{S}_2^\dagger \hat{\Pi}(\beta)\hat{S}_2 = \frac{1}{2}\Big[\hat{\Pi}(\beta-\alpha) + \hat{\Pi}(-\beta-\alpha) - \mathrm{e}^{\mathrm{i}(\phi+4\mathrm{Im}\,\{\alpha^*\beta\})}\hat{D}(2\beta) - \mathrm{e}^{-\mathrm{i}(\phi+4\mathrm{Im}\,\{\alpha^*\beta\})}\hat{D}(-2\beta)\Big] \tag{S59}$$

and therefore

$$W_2(\beta) = \frac{1}{2}\left[W_0(\beta-\alpha) + W_0(-\beta-\alpha) - \frac{4}{\pi}\mathrm{Re}\,\left\{\mathrm{e}^{\mathrm{i}(4\mathrm{Im}\,\{\alpha^*\beta\}+\phi)}\chi_0(2\beta)\right\}\right]. \tag{S60}$$

Here we have identified the characteristic function (*20*) of the initial state

$$\chi_0(\beta) \equiv \mathrm{Tr}\,\{\hat{D}(\beta)\hat{\rho}_0\} = \int \mathrm{d}^2\gamma\, \mathrm{e}^{2\mathrm{i}\mathrm{Im}\,\{\beta\gamma^*\}}W_0(\gamma). \tag{S61}$$

Note that these Wigner functions are normalized because we have implicitly assumed an $|\alpha|$ large enough such that there is no overlap between the first two terms in each of Eqs. (S60) and (S57) (Section S2.1). For these values of $|\alpha|$, the third term in each equation oscillates rapidly enough to integrate to zero.

We now specialize to the thermal state, i.e. we set $\hat{\rho}_0 = \hat{\rho}_T$. The Wigner function of the thermal initial state is

$$W_T(\beta) = \frac{2\mathcal{P}}{\pi}\mathrm{e}^{-2\mathcal{P}|\beta|^2} \tag{S62}$$

where $\mathcal{P} = (2n_{\mathrm{th}} + 1)^{-1}$ is the purity of the thermal state. The corresponding characteristic function is

$$\chi_T(\beta) = \mathrm{e}^{-|\beta|^2/2\mathcal{P}}. \tag{S63}$$

Replacing $W_0(\beta)$ and $\chi_T(\beta)$ in Eq. (S60) and Eq. (S57) gives

$$W_1(\beta) = \frac{\mathcal{P}}{\pi}\left[\mathrm{e}^{-2\mathcal{P}|\beta-\alpha|^2} + \mathrm{e}^{-2\mathcal{P}|\beta+\alpha|^2} - 2\cos\left(4\mathrm{Im}\,\{\alpha^*\beta\} + \phi\right)\mathrm{e}^{-2\mathcal{P}|\beta|^2}\right]. \tag{S64}$$

$$W_2(\beta) = \frac{1}{\pi}\left[\mathcal{P}\left(\mathrm{e}^{-2\mathcal{P}|\beta-\alpha|^2} + \mathrm{e}^{-2\mathcal{P}|\beta+\alpha|^2}\right) - 2\cos\left(4\mathrm{Im}\,\{\alpha^*\beta\} + \phi\right)\mathrm{e}^{-2|\beta|^2/\mathcal{P}}\right]. \tag{S65}$$

These are the Wigner functions given in the main text up to a redefinition of the parameter ϕ ($\phi \to \phi + \pi$). Eq. (S64) is also obtained from $W_1'(\beta)$ if one inserts the thermal Wigner function and redefines ϕ to absorb the geometric phase.

S2.3 Hot cat state coherence functions

In this section, we give the full two-dimensional hot cat coherence functions as well as their derivations. As stated in the main text, the first-order coherence function of a quantum state with density matrix $\hat{\rho}$ is defined as

$$g(x_1, x_2) \equiv \frac{|\langle x_1|\hat{\rho}|x_2\rangle|}{\sqrt{\langle x_1|\hat{\rho}|x_1\rangle\langle x_2|\hat{\rho}|x_2\rangle}}. \tag{S66}$$

Here $x_{1,2}$ are real-valued dimensionless numbers, and $|x_{1,2}\rangle$ are eigenkets of the quadrature operator

$$\hat{x} \equiv \frac{\hat{c}+\hat{c}^\dagger}{\sqrt{2}}, \tag{S67}$$

meaning that $\hat{x}|x_1\rangle = x_1|x_1\rangle$ and equivalently for x_2. Due to the positive semidefiniteness of $\hat{\rho}$, the coherence function is bounded: $1 \geq g(x_1, x_2) \geq 0$.

We compute the coherence function from $W_{1,2}(\beta)$ using the relation

$$\langle x_1|\hat{\rho}|x_2\rangle = \frac{1}{2}\int_{-\infty}^{\infty} \mathrm{d}p\, W\left(\frac{x_1+x_2}{2\sqrt{2}} + \frac{\mathrm{i}p}{\sqrt{2}}\right) \mathrm{e}^{\mathrm{i}p(x_1-x_2)} \tag{S68}$$

which can be derived e.g. from the expression for computing expectation values from the Wigner function $\langle\hat{A}\rangle = \mathrm{Tr}\{\hat{A}\hat{\rho}\} = \int \mathrm{d}^2\gamma\, 2\mathrm{Tr}\{\hat{\Pi}(\gamma)\hat{A}\}W(\gamma)$ with $\hat{A} = |x_2\rangle\langle x_1|$. It is helpful to first remind ourselves of the coherence function of a thermal state. Using Eq. (S62) in Eq. (S68), we find the coherence function

$$g_T(x_1, x_2) \equiv \exp\left\{-\frac{(x_1-x_2)^2}{4}\frac{(1-\mathcal{P}^2)}{\mathcal{P}}\right\} \tag{S69}$$

which we denote g_T since it is the coherence function of the thermal state $\hat{\rho}_T$. The thermal state coherence function is independent of the position on the diagonal $x_1 + x_2$ and is a Gaussian in the distance from the diagonal $x_1 - x_2$. Its standard deviation

$$\xi_{\mathrm{th}} \equiv \sqrt{\frac{2\mathcal{P}}{1-\mathcal{P}^2}}. \tag{S70}$$

is termed the coherence length. When $\mathcal{P}^2 \ll 1$, $\xi_{\mathrm{th}} \approx \sqrt{2\mathcal{P}}$. In this case, the coherence length is related to the quadrature standard deviation of the thermal state $\sigma_x \equiv \sqrt{\mathrm{Tr}\{\hat{x}^2\hat{\rho}_0\}} = 1/\sqrt{2\mathcal{P}}$ by the reciprocal relationship $\xi_{\mathrm{th}} = 1/\sigma_x$. In the opposite limit, $\mathcal{P} \to 1$, $\xi_{\mathrm{th}} \to \infty$ since in this case $g(x_1, x_2) \to 1$.

We denote the coherence functions of the ECD and qcMAP states respectively as $g_1(x_1, x_2)$ and $g_2(x_1, x_2)$. We also introduce the notation $\bar{x} \equiv (x_1 + x_2)/2$ and $\Delta x \equiv x_1 - x_2$ to make expressions more concise. From Eq. (S66), Eq. (S68) and Eqs. (S65) and (S64), we compute

$$g_1(x_1,x_2) = \frac{\mathrm{e}^{-(\Delta x)^2/2\xi_{\mathrm{th}}^2}\left|\cosh\left(\sqrt{2}\alpha\mathcal{P}2\bar{x}\right) - \mathrm{e}^{-4\alpha^2/\xi_{\mathrm{th}}^2}\cosh\left(\sqrt{2}\alpha\Delta x/\mathcal{P} - \mathrm{i}\phi\right)\right|}{\sqrt{\left[\cosh\left(2\sqrt{2}\alpha\mathcal{P}x_1\right) - \mathrm{e}^{-4\alpha^2/\xi_{\mathrm{th}}^2}\cos(\phi)\right]\left[\cosh\left(2\sqrt{2}\alpha\mathcal{P}x_2\right) - \mathrm{e}^{-4\alpha^2/\xi_{\mathrm{th}}^2}\cos(\phi)\right]}}. \tag{S71}$$

and

$$g_2(x_1,x_2) = \frac{\left|\mathrm{e}^{-(\Delta x)^2/2\xi_{\mathrm{th}}^2}\cosh\left(\sqrt{2}\alpha\mathcal{P}2\bar{x}\right) - \mathrm{e}^{-(2\bar{x})^2/2\xi_{\mathrm{th}}^2}\cosh\left(\sqrt{2}\alpha\mathcal{P}\Delta x - \mathrm{i}\phi\right)\right|}{\sqrt{\left[\cosh(2\sqrt{2}\alpha\mathcal{P}x_1) - \mathrm{e}^{-2x_1^2/\xi_{\mathrm{th}}^2}\cos(\phi)\right]\left[\cosh(2\sqrt{2}\alpha\mathcal{P}x_2) - \mathrm{e}^{-2x_2^2/\xi_{\mathrm{th}}^2}\cos(\phi)\right]}} \tag{S72}$$

These expressions are exact, and we plot them as 2-dimensional functions of x_1 and x_2 in Figure S8. In the remainder of this section, we will explain the appearance of Figure S8 by making approximations.

We are mainly interested in understanding $g_{1,2}$ for $\mathcal{P} \ll 1$ and for (x_1, x_2) away from the origin $(0, 0)$ (since our nonzero entries in $\langle x_1|\hat{\rho}|x_2\rangle$ are centered around the four points $(\pm\sqrt{2}\alpha, \pm\sqrt{2}\alpha)$, $(\pm\sqrt{2}\alpha, \mp\sqrt{2}\alpha)$, and we must have $\alpha > \sigma_x = 1/\sqrt{2\mathcal{P}}$). When the purity is not close to 1, the terms $\propto \cos(\phi)$ in the denominators of $g_{1,2}$ are generally negligible (g_1) or nonzero only close to the coordinate axes (g_2). We therefore drop these terms, which makes the denominators of $g_{1,2}$ equal. Using the hyperbolic trig relations, we rewrite the denominators to be $[\cosh(2\sqrt{2}\alpha\mathcal{P}2\bar{x}) + \cosh(2\sqrt{2}\alpha\mathcal{P}\Delta x)]/2$. Finally, we approximate $\cosh(x) \approx \exp(|x|)/2$ for both $\bar{x}$ and Δx. This gives

$$g_1(x_1, x_2) \approx \frac{\exp\left\{-(\Delta x)^2/2\xi_{\text{th}}^2\right\}}{\sqrt{1 + \exp\left\{2\sqrt{2}\alpha\mathcal{P}(|\Delta x| - 2|\bar{x}|)\right\}}} + \frac{\exp\left\{-(|\Delta x| - 2\sqrt{2}\alpha)^2/2\xi_{\text{th}}^2\right\}}{\sqrt{1 + \exp\left\{2\sqrt{2}\alpha\mathcal{P}(2|\bar{x}| - |\Delta x|)\right\}}}. \tag{S73}$$

$$g_2(x_1, x_2) \approx \frac{\exp\left\{-(\Delta x)^2/2\xi_{\text{th}}^2\right\}}{\sqrt{1 + \exp\left\{2\sqrt{2}\alpha\mathcal{P}(|\Delta x| - 2|\bar{x}|)\right\}}} + \frac{\exp\left\{-(2\bar{x})^2/2\xi_{\text{th}}^2\right\}}{\sqrt{1 + \exp\left\{2\sqrt{2}\alpha\mathcal{P}(2|\bar{x}| - |\Delta x|)\right\}}}, \tag{S74}$$

These expressions differ only in the nominator of the last term. The denominators are approximately indicator functions for the $x_1x_2 > 0$ quadrants (first term denominator) and $x_1x_2 < 0$ quadrants (second term denominator) of the (x_1, x_2) plane, i.e.

$$\frac{1}{\sqrt{1 + \exp\left\{2\sqrt{2}\alpha\mathcal{P}(|\Delta x| - 2|\bar{x}|)\right\}}} \approx [x_1x_2 > 0] = \begin{cases} 1 & x_1x_2 > 0 \\ 0 & x_1x_2 < 0 \end{cases}, \tag{S75}$$

$$\frac{1}{\sqrt{1 + \exp\left\{2\sqrt{2}\alpha\mathcal{P}(2|\bar{x}| - |\Delta x|)\right\}}} \approx [x_1x_2 < 0] = \begin{cases} 0 & x_1x_2 > 0 \\ 1 & x_1x_2 < 0 \end{cases}. \tag{S76}$$

(The notation [ ] for the conditional expressions is called the Iverson bracket). Using this observation, we arrive at our final expressions for $g_{1,2}$:

$$g_1(x_1, x_2) \approx g_T(x_1, x_2)[x_1x_2 > 0] + g_T(|x_1 - x_2|, 2\sqrt{2}\alpha)[x_1x_2 < 0]. \tag{S77}$$

$$g_2(x_1, x_2) \approx g_T(|x_1|, |x_2|). \tag{S78}$$

When $\mathcal{P} \ll 1$, the approximation Eq. (S75) loses accuracy in Eq. (S73), and in this limit we should instead replace $[x_1x_2 > 0]$ by 1 in Eq. (S77) to get an accurate approximation for $g_1(x_1, x_2)$. Eqs. (S77) and (S78) agree well with the exact functions plotted in Figure S8.

Along the line $l(s) \equiv (x_1(s), x_2(s)) = \sqrt{2}\alpha(2s - 1, -1)$, $s \in [0, \infty]$ in the (x_1, x_2) plane, g_1 and g_2 are equal within our approximations leading up to Eqs. (S77) and (S78). This is the line along which we plot the hot cat state coherence functions in Figure 1C in the main text.

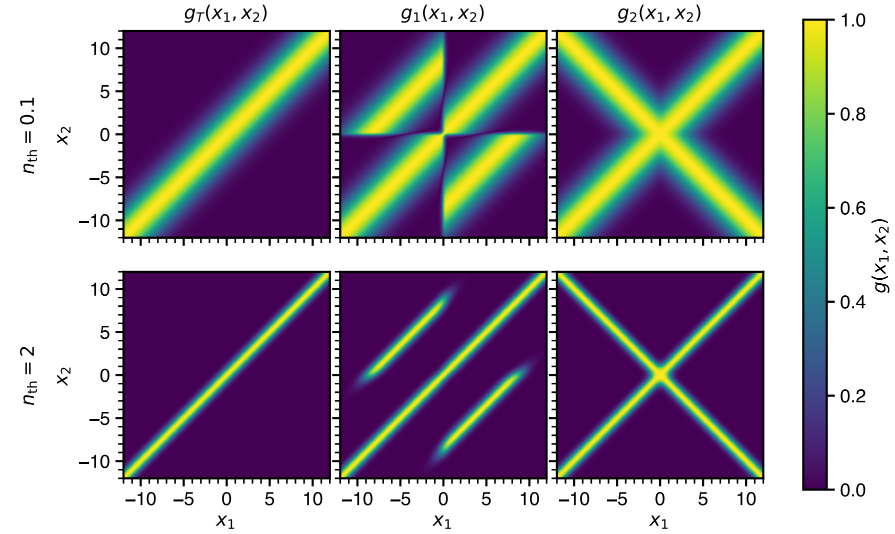

Fig. S8. Hot Schrödinger Cat State Coherence Functions. Shown is the thermal state coherence function $g_T(x_1, x_2)$ Eq. (S69) (left column), and the two cat coherence functions $g_1(x_1, x_2)$ Eq. (S71) (center column) and $g_2(x_1, x_2)$ Eq. (S72) (right column) for $n_{\rm th}$ 0.1 (top row) and 2 (bottom row). The plot uses $\alpha = 3$ and $\phi = 0$.

S3 Numerical model

Based on our characterization of the experimental setup, we introduce the numerical *ab initio* model of our experiment and compare its predictions to the experimentally measured data.

S3.1 Method

We perform all numerical work using QuTiP version 4.7 (*40*). We simulate the ECD and qcMAP protocols as follows: Displacement operators and thermal initial states are implemented using QuTiP's built-in functions. The time evolution operations $\hat{T}(t)$ are implemented using QuTiP's built-in functions to simulate the cavity-qubit dynamics during time evolution, as described in the next paragraph. The qubit and cavity-conditional qubit operations are implemented by simulating the qubit-cavity dynamics under a driving Hamiltonian, as described in more detail below. This lets us compute the density matrix resulting from the state preparation protocols. We obtain the result of the Wigner function measurement from this density matrix by computing the expectation value of the observable

$$\hat{M}(\beta) \equiv \frac{2}{\pi}\hat{\Pi}(\beta)\left(|g\rangle\langle g| - |e\rangle\langle e|\right). \tag{S79}$$

This observable gives the outcome of the Wigner function measurement when decoherence and nonlinearities during the measurement sequence are neglected (*15*).

We simulate the time-evolution operators $\hat{T}(t)$ by using QuTiP's built-in `mesolve` function to solve the following Lindblad equation

$$\frac{\partial}{\partial t}\hat{\rho}(t) = \mathcal{L}\hat{\rho}(t) \equiv -\frac{\mathrm{i}}{\hbar}\left[\hat{H},\, \hat{\rho}(t)\right] + \left(\gamma_1\mathcal{D}\left[|g\rangle\langle e|\right] + \frac{\gamma_2}{2}\mathcal{D}\left[\hat{\sigma}_z\right] + \Gamma\mathcal{D}\left[\hat{c}\right]\right)\hat{\rho}(t) \tag{S80}$$

from $t = t_0$, where $\hat{\rho}(t_0)$ is the total cavity-qubit state before the $\hat{T}$ operation is to be applied, until the final time t. The state $\hat{\rho}(t)$ is then used as input for the next step of the protocol. Here $\gamma_1 = 1/T_1$, $\gamma_2 = 1/T_2^*$ are the dissipation and dephasing rates of the qubit and $\Gamma = 1/T_{1,\mathrm{c}}$ is the dissipation rate of the cavity. $\mathcal{D}$ is the dissipator superoperator, defined as

$$\mathcal{D}[\hat{A}]\hat{\rho}(t) \equiv \hat{A}\hat{\rho}(t)\hat{A}^\dagger - \frac{1}{2}\left(\hat{A}^\dagger\hat{A}\hat{\rho}(t) + \hat{\rho}(t)\hat{A}^\dagger\hat{A}\right), \tag{S81}$$

for an arbitrary operator $\hat{A}$, and $\hat{H}$ is the Hamiltonian in the interaction picture of the cavity and qubit including the dominant higher-order perturbations

$$\frac{1}{\hbar}\hat{H} \equiv -\chi_{\mathrm{qc}}\hat{c}^\dagger\hat{c}|e\rangle\langle e| - \left(\frac{K_{\mathrm{c}}}{2} + \frac{\chi'_{\mathrm{qc}}}{2}|e\rangle\langle e|\right)\hat{c}^\dagger\hat{c}^\dagger\hat{c}\hat{c}. \tag{S82}$$

We choose the parameters $K_{\mathrm{c}}, \chi'_{\mathrm{qc}}, \gamma_1, \gamma_2, \Gamma$ to be the values measured in the experiment (Table S1).

For the qubit operations, we use QuTiP's built-in `mesolve` function to solve the Lindblad equation

$$\frac{\partial}{\partial t}\hat{\rho}(t) = \mathcal{L}\hat{\rho}(t) - \frac{\mathrm{i}}{\hbar}\left[\hat{H}_{\mathrm{drive}},\, \hat{\rho}(t)\right] \tag{S83}$$

from time t_0, where $\hat{\rho}(t_0)$ is the total cavity-qubit state before the qubit operation is applied, to time t, when the next operation is applied, and we use $\hat{\rho}(t)$ as initial state for the following operation. The driving Hamiltonian is

$$\frac{1}{\hbar}\hat{H}_{\mathrm{drive}} \equiv \frac{\Omega(t)}{2}\left(\mathrm{e}^{\mathrm{i}\phi}|e\rangle\langle g| + \mathrm{e}^{-\mathrm{i}\phi}|g\rangle\langle e|\right) \tag{S84}$$

with

$$\Omega(t) \equiv \frac{\theta \mathrm{e}^{-(t-t_0-T/2)^2/2\sigma_t^2}}{\sqrt{2\pi\sigma_t^2}\,\mathrm{erf}\left(T/2^{3/2}\sigma_t\right)}. \tag{S85}$$

Here T is the pulse duration so that $t = T + t_0$, erf is the error function, and θ is the pulse area over the interval T:

$$\int_{t_0}^{t_0+T} \mathrm{d}\tau\, \Omega(\tau) = \theta. \tag{S86}$$

We use $T = 4\sigma_t$ as this is the value used in the experiment. We take θ, σ_t and ϕ as the parameters of the pulse and choose them according to the desired operation to be modelled. In particular, $\sigma_t = 6$ ns for the global qubit operations, and $\sigma_t = 20$ ns for the cavity-selective disentanglement pulse. We compensate the free evolution times for the finite length of the qubit pulses, so that the maximum of the disentanglement pulse occurs at $t = \pi/\chi_{\mathrm{qc}}$ in both protocols, and in the ECD protocol, the maximum of the qubit echo pulse occurs at $t = \pi/2\chi_{\mathrm{qc}}$.

S3.2 Comparison of Simulation and Data

We run our simulations with all parameters of the Lindbladian taking the values that we measure experimentally (Table S1), and the pulse parameters being the experimentally used values. We present a comparison of the simulation results to the experimental Wigner map data in Figure S9. Here, we simulated with $n_{\mathrm{th}} = 3.48$, $\alpha = 3.06$ for the ECD protocol and $\alpha = 3.47$ for the qcMAP protocol, and $\phi = \pi$. To facilitate a comparison of the simulations to the data, we displace the simulated Wigner functions so that their fringe pattern aligns with that of the data. We do this by identifying the coherence fringe in the simulated Wigner functions corresponding most closely to the coherence fringe centered at $\beta = 0$ in the data. We then displace the simulated Wigner functions so that the identified fringe is also centered at $\beta = 0$. Specifically, the simulated ECD Wigner function was displaced by $0.654\mathrm{i}$ and the simulated qcMAP Wigner function was displaced by $0.111 - 0.353\mathrm{i}$. We additionally rotate the qcMAP state clockwise by 0.163 radians so that the centers of the displaced thermal states lie along the $\mathrm{Re}\{\beta\}$ axis. The ECD state was not rotated.

Using the same simulation parameters as for the Wigner maps (including the final displacement and rotations), we also simulate the Wigner function linecuts along the $\mathrm{Re}\left\{\beta\right\}$ and $\mathrm{Im}\left\{\beta\right\}$ axes for the values of n_{th} that were reported in Figure 2D-G. We present the computed linecuts in Figure S10.

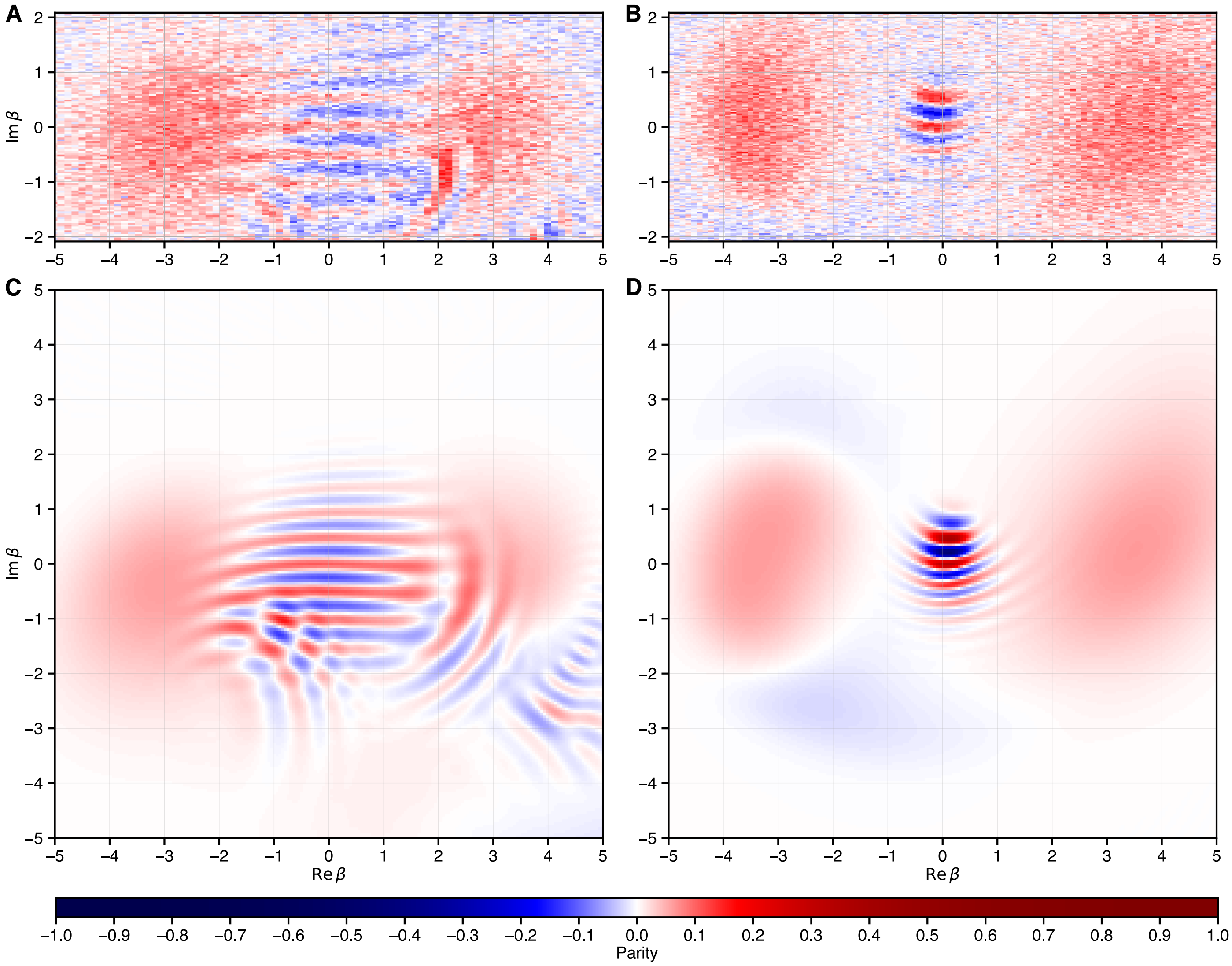

Fig. S9. Comparison of simulated Wigner maps to measured data. (**A**) Experimental data obtained for the ECD protocol (also displayed in Figure 1B). (**B**) Experimental data obtained for the qcMAP protocol (also displayed in Figure 1C). (**C**) Result of the numerical simulation of the ECD protocol. (**D**) Result of the numerical simulation of the qcMAP protocol.

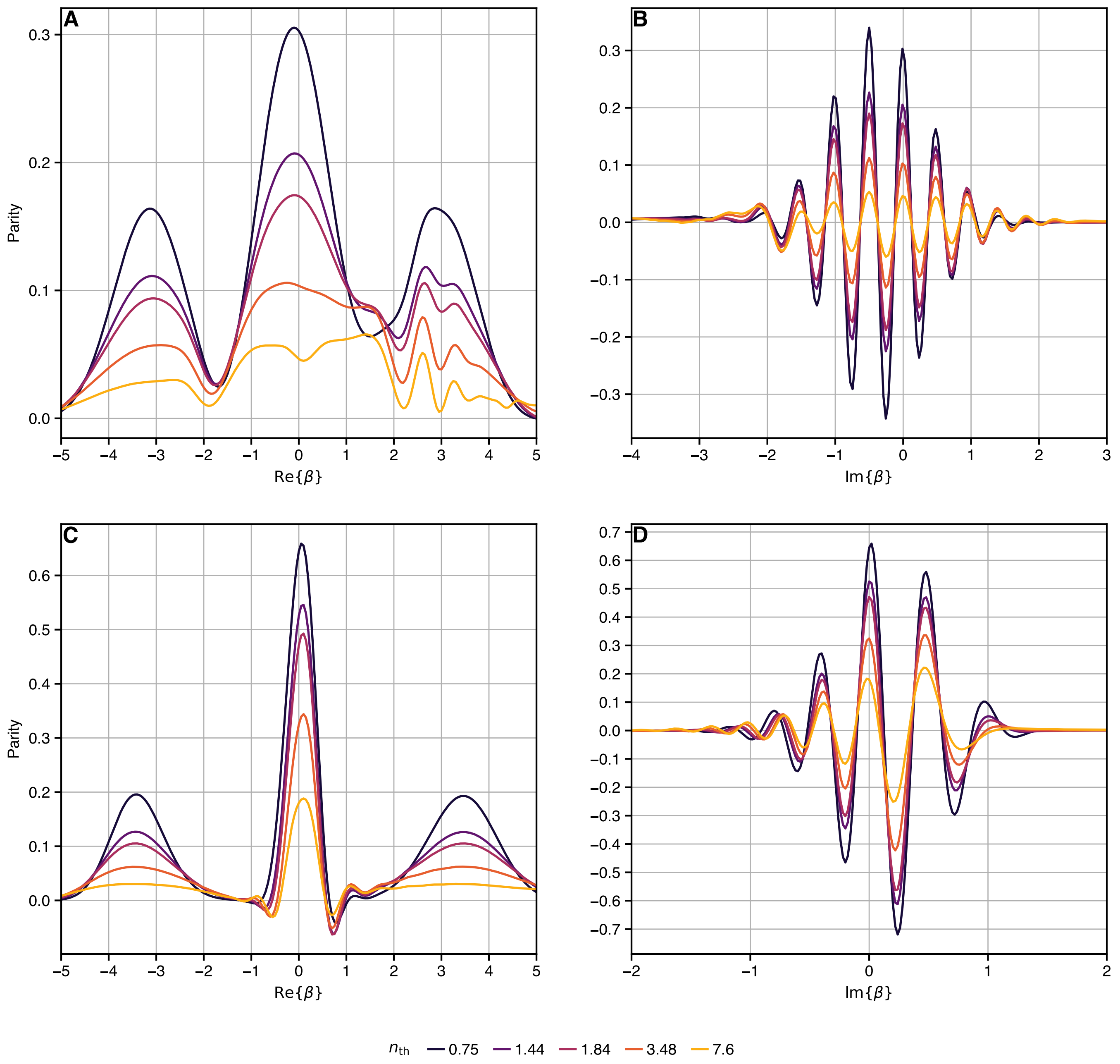

Fig. S10. Wigner function linecuts obtained from simulation. The line colors are chosen in analogy to the line colors in Figure 1D-G. (**A**) Linecut along $\mathrm{Re}\{\beta\}$ through the ECD Wigner function. (**B**) Linecut along $\mathrm{Im}\{\beta\}$ through the ECD Wigner function. (**C**) Linecut along $\mathrm{Re}\{\beta\}$ through the qcMAP Wigner function. (**D**) Linecut along $\mathrm{Im}\{\beta\}$ through the qcMAP Wigner function.

S3.3 Cavity phase noise

As discussed in the main text and in Section S1.7, the experiment has additional loss channels not included in the numerical model, such as cavity phase noise. In Figure 4 of the main text, we have illustrated the effect of adding this noise to the model by adding a term $\frac{\Gamma_{\phi,c}}{2}\mathcal{D}[\hat{n}]\hat{\rho}$ to Eq. (S80). The dotted curves in Figure 4 are the results of running the numerical model with $\Gamma_{\phi,c} = 1/(80\ \mu\text{s})$. We stress that this value has been chosen to illustrate the effect, the exact dephasing rate has to be determined in additional experiments. Cavity phase noise is included only in the dotted curves in Figure 4 and nowhere else in this work.

S4 Analysis of Imperfections

The experimentally achieved protocols differ from the ideal ECD and qcMAP protocols analyzed theoretically in Section S2. In this section, we study the identified differences in isolation using a combination of analytical and numerical methods. By comparing the results to the ideal protocols and the data, we can attribute features in the data which are not seen in the theoretical Wigner functions to particular experimental imperfections.

S4.1 Residual cavity-qubit entanglement

The experimental imperfections lead to a finite probability p_e of the qubit being in the excited state at the end of the protocol. From Eq. (S79) and Eq. (S44), one computes that the expected result of the measurement is

$$W_{\text{meas.}}(\beta) \equiv \text{Tr}\left\{\hat{M}(\beta)\hat{U}\hat{\rho}_0|g\rangle\langle g|\hat{U}^\dagger\right\} = p_g W_g(\beta) - p_e W_e(\beta). \tag{S87}$$

Here, we have introduced the qubit-conditional Wigner functions

$$W_{g,e}(\beta) \equiv \frac{2}{\pi}\text{Tr}\,\{\hat{\Pi}(\beta)\hat{\rho}_{g,e}\}. \tag{S88}$$

Imperfect disentanglement between the cavity and qubit will lead to $p_e > 0$, $p_g < 1$. In this case, the output of the Wigner function measurement is not the final state Wigner function, but rather the weighted difference of the Wigner functions of the qubit-conditional states $\hat{\rho}_g$ and $\hat{\rho}_e$, with the weights being p_g and p_e. As long as $p_g \gg p_e$ and/or the Wigner functions $W_g(\beta)$ and $W_e(\beta)$ do not overlap significantly, the residual qubit-cavity entanglement is a perturbative imperfection to measurement of the final cavity state Wigner function.

S4.2 Comparison of simulations with different parameters

To understand the effect of each difference between our experiment and the ideal ECD and qcMAP protocols, we run our simulations with different sets of parameters which are chosen

to isolate each difference. We present the results of these simulations in Figures S11 (ECD) and S12 (qcMAP). We go through the parameters used for each panel of these figures in the next paragraphs.

'Experimental parameters' (panel A) refers to the simulations as described in Section S3, where we chose the parameters to match the experiment as closely as possible (however, in Figures S11 and S12, we do not rotate or displace the Wigner functions before plotting).

'Reference parameters' (panel B) refers to a set of parameters chosen to match the ideal ECD and qcMAP protocols considered in Section S2.1. These simulations have no Kerr nonlinearities, negligible width of the non-selective qubit pulses, infinite coherence times, and a disentanglement pulse width which was optimized by sweeping σ_t for these parameters and choosing the σ_t that minimized p_e in this scenario. Specifically, the reference parameters are: $K_{\mathrm{c}} = \chi'_{\mathrm{qc}} = 0$, $\Gamma = \gamma_1 = \gamma_2 = 0$, disentanglement pulse $\sigma_t = 6$ ns, other qubit pulses $\sigma_t = 10^{-13}$ s.

Panels C-F show simulations using the reference parameters but with a subset of the parameters set to their experimental values, as explained in the figure captions.

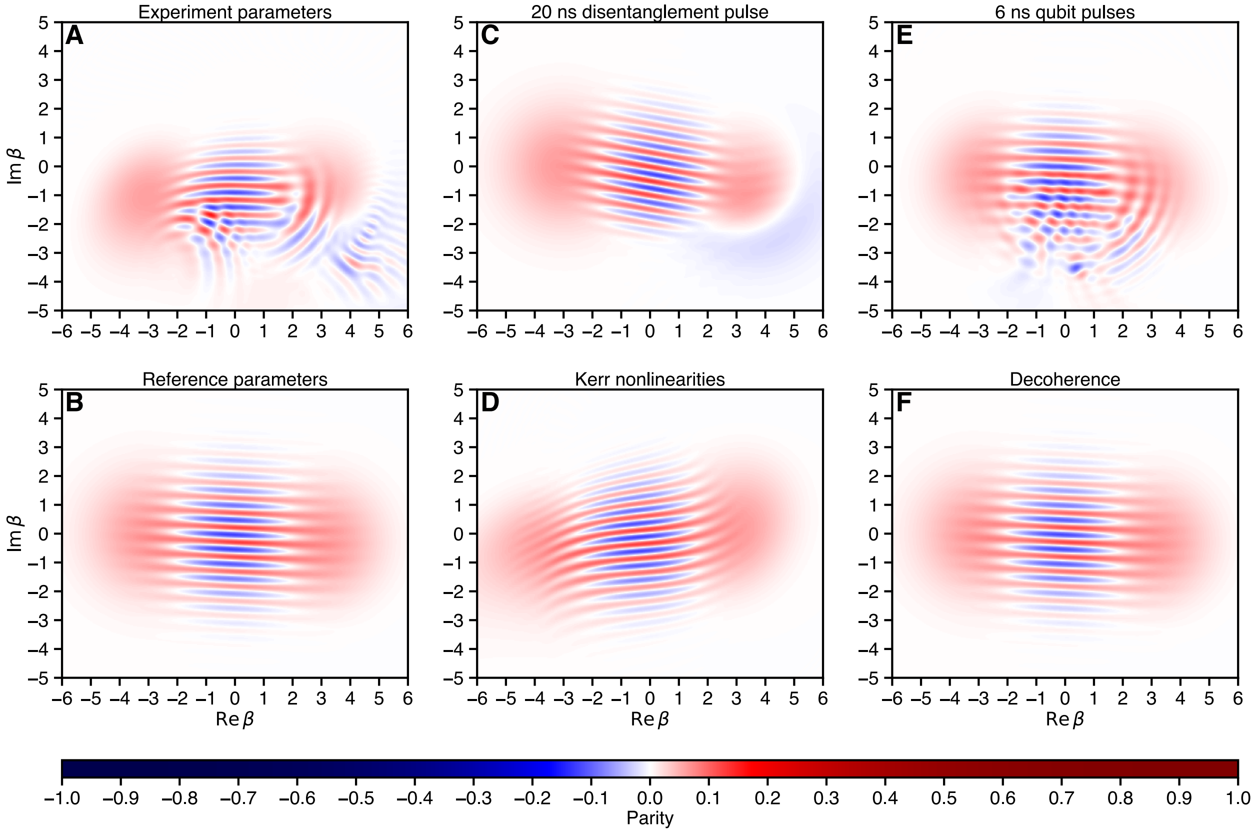

Fig. S11. Comparison of simulations with different parameters for ECD. We simulate the hot cat state preparation protocols with different sets of parameters, which are chosen to isolate the differences between our experiment and the ideal ECD protocol. All simulations shown used $n_{\mathrm{th}} = 3.48$ and $\alpha = 3.06$. (**A**) Simulation with all parameters taking the values measured or used in the experiment, as in Fig. S9C. (**B**) Simulation with parameters chosen to match the ideal ECD protocol (see Section S4.2). (**C**) Simulation with the reference parameters but the disentanglement pulse standard deviation σ_t set to the experiment value of 20 ns. (**D**) Simulation with the reference parameters but the Kerr nonlinearity parameters K_{c} and χ'_{qc} taking their experimentally measured values. (**E**) Simulation with the reference parameters but the σ_t of all qubit pulses set to 6 ns, the minimum pulse σ_t that our experimental instrumentation can achieve. (**F**) Simulation with the reference parameters but with the coherence times T_1, T_2^* and $T_{1,\mathrm{c}}$ taking their experimental values.

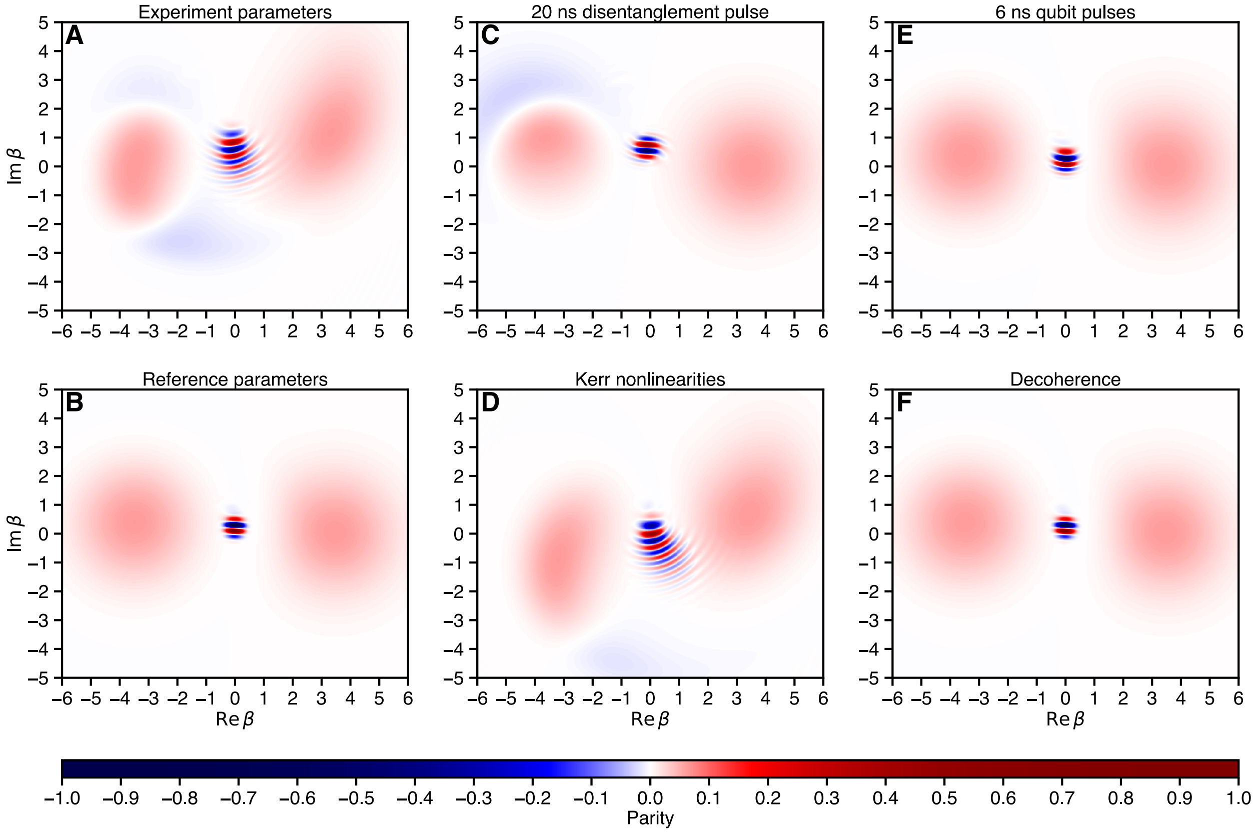

Fig. S12. Comparison of simulations with different parameters for qcMAP. We simulate the hot cat state preparation protocols with different sets of parameters, which are chosen to isolate the differences between our experiment and the ideal qcMAP protocol. All simulations shown used $n_{\rm th} = 3.48$ and $\alpha = 3.47$. (**A**) Simulation with all parameters taking the values measured or used in the experiment, as in Fig. S9D. (**B**) Simulation with parameters chosen to match the ideal qcMAP protocol (see Section S4.2). (**C**) Simulation with the reference parameters but the disentanglement pulse standard deviation σ_t set to the experiment value of 20 ns. (**D**) Simulation with the reference parameters but the Kerr nonlinearity parameters $K_{\rm c}$ and $\chi'_{\rm qc}$ taking their experimentally measured values. (**E**) Simulation with the reference parameters but the σ_t of all qubit pulses set to 6 ns, the minimum pulse σ_t that our experimental instrumentation can achieve. (**F**) Simulation with the reference parameters but with the coherence times T_1, T_2^* and $T_{1,\rm c}$ taking their experimental values.

S4.3 Free evolution timing errors

In the experiment, all cavity and qubit operation pulses take a finite time. Cavity displacement pulses take 16 ns, and qubit operations take $4\sigma_t$ to complete (24 ns for non-selective qubit pulses, 80 ns for the disentanglement pulse). The design of the experimental protocol takes this into account by optimizing the pulse timings, with a step size of 4 ns. Nevertheless, here we theoretically investigate the effects of perturbing the time argument of the free evolution operator $\hat{T}(t)$ in the qcMAP protocol. We show that such perturbations, if present, lead to bending distortions of the hot cat state fringes which are not seen for cold cat states.

In the ideal qcMAP protocol, the free evolution time is π/χ_{qc}. Here we take the free evolution time to be $\pi/\chi_{\mathrm{qc}} + \tau$, where τ is the timing error. Following the analysis in Section S2.1, the effective operator of the protocol becomes

$$\hat{S}_1 = \frac{1}{\sqrt{2}}\left[1 - \exp\left\{\mathrm{i}\left(\chi_{\mathrm{qc}}\tau\hat{n} + \phi\right)\right\}\hat{\Pi}\right]\hat{D}(\alpha). \tag{S89}$$

The Wigner function resulting from the application of this operator on a cavity state can be computed using the same methods as in Section S2.2. The result is

$$W_1(\beta) = \frac{1}{2}\Bigg[W_0(\beta-\alpha) + W_0(-\alpha-\beta\mathrm{e}^{-\mathrm{i}\chi_{\mathrm{qc}}\tau}) - \frac{4}{\pi}\mathrm{Re}\left\{\mathrm{e}^{\mathrm{i}\varphi}\mathrm{Tr}\left\{\hat{D}\left[2\beta+\alpha(\mathrm{e}^{\mathrm{i}\chi_{\mathrm{qc}}\tau}-1)\right]\exp\left\{\mathrm{i}\chi_{\mathrm{qc}}\tau\hat{n}\right\}\hat{\rho}_0\right\}\right\}\Bigg] \tag{S90}$$

with

$$\varphi = \phi + 2\mathrm{Im}\left\{\alpha^*\beta\left[1+\exp\left(-\mathrm{i}\chi_{\mathrm{qc}}\tau\right)\right]\right\} + |\alpha|^2\sin\left(\chi_{\mathrm{qc}}\tau\right). \tag{S91}$$

For a thermal state $\hat{\rho}_0 = \hat{\rho}_T$,

$$\mathrm{Tr}\left\{\hat{D}\left[2\beta+\alpha(\mathrm{e}^{\mathrm{i}\chi_{\mathrm{qc}}\tau}-1)\right]\exp\left\{\mathrm{i}\chi_{\mathrm{qc}}\tau\hat{n}\right\}\hat{\rho}_0\right\} = \\ = \frac{1}{1+n_{\mathrm{th}}\left(1-\mathrm{e}^{\mathrm{i}\chi_{\mathrm{qc}}\tau}\right)}\exp\left\{-\left(\frac{1}{2}+\frac{n_{\mathrm{th}}\mathrm{e}^{\mathrm{i}\chi_{\mathrm{qc}}\tau}}{1+n_{\mathrm{th}}\left(1-\mathrm{e}^{\mathrm{i}\chi_{\mathrm{qc}}\tau}\right)}\right)\left|2\beta+\alpha\left(\mathrm{e}^{\mathrm{i}\chi_{\mathrm{qc}}\tau}-1\right)\right|^2\right\}. \tag{S92}$$

Taking $\tau = 0$ recovers Eq. (S65) as expected. To understand the effect of a small τ, we linearize Eq. (S91) and Eq. (S92) in τ. Taking α to be real, the coherence term in Eq. (S90) linearized in τ is

$$\frac{4}{\pi}\cos\left\{4\alpha\mathrm{Re}\left\{k^*\beta\right\} + \phi' + 4\chi_{\mathrm{qc}}\tau|\beta|^2 n_{\mathrm{th}}(1+n_{\mathrm{th}})\right\} \cdot \exp\left\{-2(2n_{\mathrm{th}}+1)\left(|\beta|^2 + \chi_{\mathrm{qc}}\tau\alpha\mathrm{Im}\left\{\beta\right\}\right)\right\}. \tag{S93}$$

Here $k \equiv -\chi_{\mathrm{qc}}\tau/2 + \mathrm{i}$ and $\phi' \equiv \phi + \chi_{\mathrm{qc}}\tau(|\alpha|^2 + n_{\mathrm{th}})$. This expression contains three additional effects compared to the $\tau = 0$ case: 1) The phase shift has changed from ϕ to ϕ'. 2) Since

the left Gaussian has moved in the $\mathrm{Im}\{\beta\}$ direction, the center of the fringes has also moved in the $\mathrm{Im}\{\beta\}$ direction, and k has obtained a real part. 3) The presence of $|\beta|^2$ in the cosine argument causes a bending distortion of the fringes. The $|\beta|^2$ term vanishes when $n_{\mathrm{th}} \to 0$, so that noticeable bending of the fringes occurs only for hot cats.

For illustration, we plot Eq. (S90) in Figure S13 for $\alpha = 3.47$, $n_{\mathrm{th}} = 3.48$, $\phi = \pi$, and a timing error of $\tau = 20$ ns. We emphasize that this value of τ is much larger than the 4 ns steps with which we optimize the pulse timings in our experiment. Figure S13 bears some resemblance to Figures S9 and S10; however, our numerical simulations do not contain any timing error, and the fringe bending observed in our experiment is mainly due to the Kerr nonlinearity (see Section S4.2).

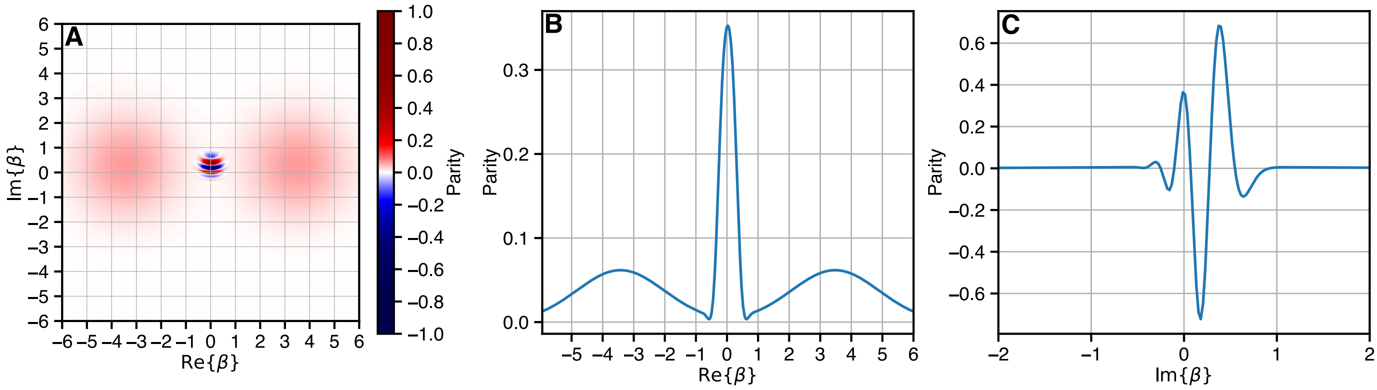

Fig. S13. Fringe bending due to timing errors. (**A**) Wigner function Eq. (S90) for a timing error of 20 ns. The Wigner function has been rotated and displaced before plotting to remove the extra rotation due to the timing error and center the fringe pattern at $\beta = 0$. (**B**) Linecut through the Wigner function in panel A along $\mathrm{Re}\,\beta$. (**C**) Linecut through the Wigner function in panel A along $\mathrm{Im}\,\beta$.

REFERENCES AND NOTES

1. R. J. Glauber, Coherent and incoherent states of the radiation field. *Phys. Rev.* **131**, 2766–2788 (1963).

2. E. Schrödinger, Die gegenwärtige situation in der Quantenmechanik. *Naturwissenschaften* **23**, 807–812 (1935).

3. C. Monroe, D. M. Meekhof, B. E. King, D. J. Wineland, A "Schrödinger cat" superposition state of an atom. *Science* **272**, 1131–1136 (1996).

4. M. Brune, E. Hagley, J. Dreyer, X. Maître, A. Maali, C. Wunderlich, J.M. Raimond, S. Haroche, Observing the progressive decoherence of the "meter" in a quantum measurement. *Phys. Rev. Lett.* **77**, 4887–4890 (1996).

5. K. Zhu, H. Tang, C. Li, D. Huang, X. Li, X. Li, The quantum statistical properties of superposition coherent states with thermal noise. *J. Mod. Opt.* **43**, 323–336 (1996).

6. G. Huyet, S. Franke-Arnold, S. M. Barnett, Superposition states at finite temperature. *Phys. Rev. A* **63**, 043812 (2001).

7. H. Jeong, T. C. Ralph, Transfer of nonclassical properties from a microscopic superposition to macroscopic thermal states in the high temperature limit. *Phys. Rev. Lett.* **97**, 100401 (2006).

8. S.-B. Zheng, Macroscopic superposition and entanglement for displaced thermal fields induced by a single atom. *Phys. Rev. A* **75**, 032114 (2007).

9. H. Jeong, T. C. Ralph, Quantum superpositions and entanglement of thermal states at high temperatures and their applications to quantum-information processing. *Phys. Rev. A* **76**, 042103 (2007).

10. F. Nicacio, R. N. P. Maia, F. Toscano, R. O. Vallejos, Phase space structure of generalized Gaussian cat states. *Phys. Lett. A* **374**, 4385–4392 (2010).

11. A. Eickbusch, V. Sivak, A. Z. Ding, S. S. Elder, S. R. Jha, J. Venkatraman, B. Royer, S. M. Girvin, R. J. Schoelkopf, M. H. Devoret, Fast universal control of an oscillator with weak dispersive coupling to a qubit. *Nat. Phys.* **18**, 1464–1469 (2022).

12. Z. Leghtas, G. Kirchmair, B. Vlastakis, M. H. Devoret, R. J. Schoelkopf, M. Mirrahimi, Deterministic protocol for mapping a qubit to coherent state superpositions in a cavity. *Phys. Rev. A* **87**, 042315 (2013).

13. M. Reagor, W. Pfaff, C. Axline, R. W. Heeres, N. Ofek, K. Sliwa, E. Holland, C. Wang, J. Blumoff, K. Chou, M. J. Hatridge, L. Frunzio, M. H. Devoret, L. Jiang, R. J. Schoelkopf, Quantum memory with millisecond coherence in circuit QED. *Phys. Rev. B* **94**, 014506 (2016).

14. P. Heidler, C. M. F. Schneider, K. Kustura, C. Gonzalez-Ballestero, O. Romero-Isart, G. Kirchmair, Non-markovian effects of two-level systems in a niobium coaxial resonator with a single-photon lifetime of 10 milliseconds. *Phys. Rev. Appl.* **16**, 034024 (2021).

15. B. Vlastakis, G. Kirchmair, Z. Leghtas, S.E. Nigg, L. Frunzio, S. M. Girvin, M. Mirrahimi, M. H. Devoret, R. J. Schoelkopf, Deterministically encoding quantum information using 100-photon Schrödinger cat states. *Science* **342**, 607–610 (2013).

16. A. Royer, Wigner function as the expectation value of a parity operator. *Phys. Rev. A* **15**, 449–450 (1977).

17. D. I. Schuster, A. A. Houck, J. A. Schreier, A. Wallraff, J. M. Gambetta, A. Blais, L. Frunzio, J. Majer, B. Johnson, M. H. Devoret, S. M. Girvin, R. J. Schoelkopf, Resolving photon number states in a superconducting circuit. *Nature* **445**, 515–518 (2007).

18. G. F. Thomas, Validity of the Rosen-Zener conjecture for Gaussian-modulated pulses. *Phys. Rev. A* **27**, 2744–2746 (1983).

19. I. S. Mihov, N. V. Vitanov, Pulse shape effects in qubit dynamics demonstrated on an IBM quantum computer. *Phys. Rev. A* **108**, 042604 (2023).

20. S. M. Barnett, P. M. Radmore, *Methods in Theoretical Quantum Optics* (Clarendon Press, 2002).

21. A. Grimm, N. E. Frattini, S. Puri, S. O. Mundhada, S. Touzard, M. Mirrahimi, S. M. Girvin, S. Shankar, M. H. Devoret, Stabilization and operation of a Kerr-cat qubit. *Nature* **584**, 205–209 (2020).

22. R. Lescanne, M. Villiers, T. Peronnin, A. Sarlette, M. Delbecq, B. Huard, T. Kontos, M. Mirrahimi, Z. Leghtas, Exponential suppression of bit-flips in a qubit encoded in an oscillator. *Nat. Phys.* **16**, 509–513 (2020).

23. R. J. Glauber, The quantum theory of optical coherence. *Phys. Rev.* **130**, 2529–2539 (1963).

24. D. E. Miller, J. R. Anglin, J. R. Abo-Shaeer, K. Xu, J. K. Chin, W. Ketterle, High-contrast interference in a thermal cloud of atoms. *Phys. Rev. A* **71**, 043615 (2005).

25. Y. Margalit, Z. Zhou, S. Machluf, Y. Japha, S. Moukouri, R. Folman, Analysis of a high-stability Stern–Gerlach spatial fringe interferometer. *New J. Phys.* **21**, 073040 (2019).

26. I. Bloch, T. W. Hänsch, T. Esslinger, Measurement of the spatial coherence of a trapped bose gas at the phase transition. *Nature* **403**, 166–170 (2000).

27. S. Deléglise, I. Dotsenko, C. Sayrin, J. Bernu, M. Brune, J.-M. Raimond, S. Haroche, Reconstruction of non-classical cavity field states with snapshots of their decoherence. *Nature* **455**, 510–514 (2008).

28. G. Kirchmair, B. Vlastakis, Z. Leghtas, S. E. Nigg, H. Paik, E. Ginossar, M. Mirrahimi, L. Frunzio, S. M. Girvin, R. J. Schoelkopf, Observation of quantum state collapse and revival due to the single-photon Kerr effect. *Nature* **495**, 205–209 (2013).

29. O. Milul, B. Guttel, U. Goldblatt, S. Hazanov, L.M. Joshi, D. Chausovsky, N. Kahn, E. Çiftyürek, F. Lafont, S. Rosenblum, Superconducting cavity qubit with tens of milliseconds single-photon coherence time. *PRX Quantum* **4**, 030336 (2023).

30. Y. Margalit, O. Dobkowski, Z. Zhou, O. Amit, Y. Japha, S. Moukouri, D. Rohrlich, A. Mazumdar, S. Bose, C. Henkel, R. Folman, Realization of a complete Stern-Gerlach interferometer: Toward a test of quantum gravity. *Sci. Adv.* **7**, eabg2879 (2021).

31. C. Samanta, S. L. de Bonis, C. B. Møller, R. Tormo-Queralt, W. Yang, C. Urgell, B. Stamenic, B. Thibeault, Y. Jin, D. A. Czaplewski, F. Pistolesi, A. Bachtold, Nonlinear nanomechanical resonators approaching the quantum ground state. *Nat. Phys.* **19**, 1340–1344 (2023).

32. M. Gutierrez Latorre, G. Higgins, A. Paradkar, T. Bauch, W. Wieczorek, Superconducting microsphere magnetically levitated in an anharmonic potential with integrated magnetic readout. *Phys. Rev. Appl.* **19**, 054047 (2023).

33. J. Hofer, R. Gross, G. Higgins, H. Huebl, O. F. Kieler, R. Kleiner, D. Koelle, P. Schmidt, J. A. Slater, M. Trupke, K. Uhl, T. Weimann, W. Wieczorek, M. Aspelmeyer, High-Q magnetic levitation and control of superconducting microspheres at millikelvin temperatures. *Phys. Rev. Lett.* **131**, 043603 (2023).

34. J. Millen, P. Z. G. Fonseca, T. Mavrogordatos, T. S. Monteiro, P. F. Barker, Cavity cooling a single charged levitated nanosphere. *Phys. Rev. Lett.* **114**, 123602 (2015).

35. T. Delord, P. Huillery, L. Nicolas, G. Hétet, Spin-cooling of the motion of a trapped diamond. *Nature* **580**, 56–59 (2020).

36. G. P. Conangla, R. A. Rica, R. Quidant, Extending vacuum trapping to absorbing objects with hybrid paul-optical traps. *Nano Lett.* **20**, 6018–6023 (2020).

37. L. Dania, D. S. Bykov, F. Goschin, M. Teller, A. Kassid, T. E. Northup, Ultrahigh quality factor of a levitated nanomechanical oscillator. *Phys. Rev. Lett.* **132**, 133602 (2024).

38. M. Scala, M. S. Kim, G. W. Morley, P. F. Barker, S. Bose, Matter-wave interferometry of a levitated thermal nano-oscillator induced and probed by a spin. *Phys. Rev. Lett.* **111**, 180403 (2013).

39. K. Marshall, D. F. V. James, A. Paler, H.-K. Lau, Universal quantum computing with thermal state bosonic systems. *Phys. Rev. A* **99**, 032345 (2019).

40. J. R. Johansson, P. D. Nation, F. Nori, QuTiP 2: A python framework for the dynamics of open quantum systems. *Comput. Phys. Commun.* **184**, 1234–1240 (2013).

41. P. Horowitz, W. Hill, *The Art of Electronics* (Cambridge Univ. Press, ed. 2, 1989).

42. F. Diedrich, J. C. Bergquist, W. M. Itano, D. J. Wineland, Laser cooling to the zero-point energy of motion. *Phys. Rev. Lett.* **62**, 403–406 (1989).

43. S. E. Hamann, D. L. Haycock, G. Klose, P. H. Pax, I. H. Deutsch, P. S. Jessen, Resolved-sideband raman cooling to the ground state of an optical lattice. *Phys. Rev. Lett.* **80**, 4149–4152 (1998).

44. J. A. Smolin, J. M. Gambetta, G. Smith, Efficient method for computing the maximum-likelihood quantum state from measurements with additive Gaussian noise. *Phys. Rev. Lett.* **108**, 070502 (2012).

45. M. S. Kim, V. Bužek, Schrödinger-cat states at finite temperature: Influence of a finite-temperature heat bath on quantum interferences. *Phys. Rev. A* **46**, 4239–4251 (1992).
